#  Myokine SIRP**α** exacerbates kidney disease in diabetes

**DOI:** 10.1172/jci.insight.183392

**Published:** 2026-02-09

**Authors:** Jiao Wu, Elisa Russo, Daniela Verzola, Qingtian Li, Helena Zhang, Bhuvaneswari Krishnan, David Sheikh-Hamad, Zhaoyong Hu, William E. Mitch, Sandhya S. Thomas

**Affiliations:** 1 Nephrology Division, Department of Medicine, Baylor College of Medicine (BCM), Houston, Texas, USA.; 2 Nephrology Division, Department of Medicine, Università degli Studi di Genova, Genoa, Italy.; 3 Department of Pathology and Immunology, BCM, Houston, Texas, USA.; 4 Nephrology Division, Department of Medicine, Michael E. DeBakey VA Medical Center, Houston, Texas, USA.

**Keywords:** Endocrinology, Nephrology, Chronic kidney disease, Diabetes

## Abstract

Mechanisms responsible for skeletal muscle kidney crosstalk have not been defined. We have determined that a circulating mediator, signal regulatory protein α (SIRPα), impairs intracellular insulin-mediated functions. To elucidate the effect of myokine SIRPα on diabetic kidney disease (DKD), flox mice and muscle-specific (m-specific) SIRPα-KO mice were subjected to an obesity-induced model of diabetes, high-fat diet (HFD; 60%) or insulin-deficient hyperglycemia model, streptozotocin (STZ), and were subsequently exposed to anti-SIRPα monoclonal antibodies. In the obesity-induced diabetic mice, serum SIRPα increased. Genetic deletion of muscle SIRPα protected against obesity and improved intracellular insulin signaling in muscle and adipose tissue, with reduced intramuscular fat deposition when compared with *flox* mice on HFD. Moreover, *mSIRP*α-KO mice displayed enhanced kidney tubular fatty acid oxidation (FAO) expression with suppressed intraorgan triglycerides deposition, and importantly, protection against DKD. Conversely, exogenous SIRPα impaired kidney proximal tubular cell FAO, ATP production, and exacerbated fibrosis. Finally, suppressing SIRPα in skeletal muscles or treatment with anti-SIRPα monoclonal antibodies in STZ-treated mice mitigated cachexia, hyperlipidemia, kidney triglyceride deposition, and renal dysfunction in spite of significant hyperglycemia. Importantly, serum SIRPα was upregulated in patients with DKD. In conclusion, SIRPα serves as a potential biomarker and therapeutic target in DKD.

## Abstract 2

Myokine SIRPα inhibits kidney proximal tubule fatty acid (FA) oxidation while promoting diabetic kidney disease.

## Graphical abstract

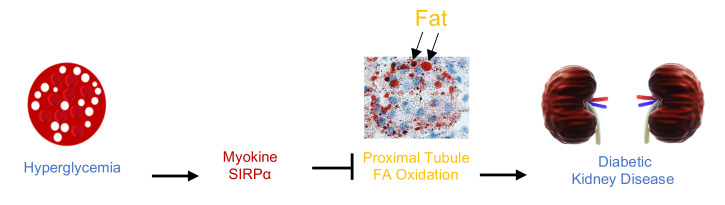

## Introduction

Chronic kidney disease (CKD) affects an estimated 15% (~37 million or 1 in 7) of US adults according to the Centers for Disease Control and Prevention (CDC) (1, 2). Patients with CKD display evidence of systemic insulin resistance with or without diabetes (3). We have determined that signal regulatory protein α (SIRPα) is responsible for postinsulin receptor defects impairing intracellular insulin signaling in a subtotal nephrectomy model of CKD (4). Specifically, SIRPα reduces tyrosine phosphorylation of insulin substrate 1 (IRS1), insulin receptor, or insulin-like growth factor-1 (IGF-1) receptor in skeletal or cardiac muscle contributing to peripheral organ insulin resistance in CKD (4, 5). Moreover, SIRPα affects insulin signaling in CKD, contributing to pathologic browning and skeletal muscle loss (6). Therefore, we examined the influence of SIRPα in diabetic kidney disease (DKD).

The muscle plays a critical role in kidney function, details of which are underrecognized. In fact, creatinine, a product of muscle tissue breakdown, is utilized for measurements of kidney function (7). Moreover, circulating myokines may be responsible for improved or worsening kidney disease. For example, myostatin has been implicated in worsening interstitial fibrosis in diabetic nephropathy (8). Additionally, exercise-associated myokines (i.e., irisin) are involved in muscle-kidney crosstalk, suppressing metabolic reprogramming and the development of kidney fibrosis (9). Skeletal muscle–specific Akt1 transgenic mice prevented skeletal muscle loss with improved kidney function despite the presence of unilateral ureteral obstruction (UUO) (10). In fact, aerobic exercise (e.g., walking) revealed benefits in cardiovascular health in predialysis patients with CKD (11–13).

Hyperglycemia alone may not correlate with worsening DKD; moreover, lower hemoglobin A1c levels < 6.5% correlated with worsening patient outcomes (14, 15). Thus, it is possible that pathologic myokines may be contributing to worsening kidney function irrespective of glucose controls. We have determined that exposure to hyperglycemia or uremic toxins stimulates SIRPα release into cultured myocyte media but not cultured adipocyte media (8). Additionally, control mice exposed to acute hyperglycemia stimulates increased serum SIRPα (8). Furthermore, patients with CKD display an increase in serum SIRPα when compared with healthy controls (8). Therefore, in this study, we examined the effects of SIRPα responses in DKD.

DKD is associated with alterations in kidney tubular FAO, but the mechanisms responsible for insulin resistance–induced impaired FAO have not been elucidated (16). FAO is a preferred mitochondrial respiratory substrate for highly metabolic cells, including cardiomyocytes and kidney proximal tubular cells (17). The kidney is second only to cardiac muscle in mitochondrial numbers and oxygen consumption (17, 18). ATP generation in the proximal tubular cells occurs mainly through fatty acid oxidation (17). Lipotoxicity has been linked to advancing kidney disease (19). Kang et al. determined that overexpression of long-chain fatty acid transporter CD36 contributed to lipid accumulation in renal tubular epithelial cells but did not directly lead to renal fibrosis (20). Therefore, the investigators concluded that lipid accumulation was not the main factor responsible for kidney dysfunction. In fact, reduced FAO or fatty acid utilization has been linked to the development of tubulointerstitial fibrosis via TGF-β1 (20). Specifically, impaired FAO induces ATP depletion, tubular necrosis, maladaptive repair, and ultimately kidney dysfunction (20). Here we examined the effect of a mediator of insulin signaling, myokine SIRPα, on kidney FAO and the importance of muscle-kidney crosstalk in DKD.

## Results

### Obesity-induced hyperglycemia stimulates serum SIRPα, while suppression of SIRPα initiates salutary effects on diet-induced obesity.

**Figure 1:**
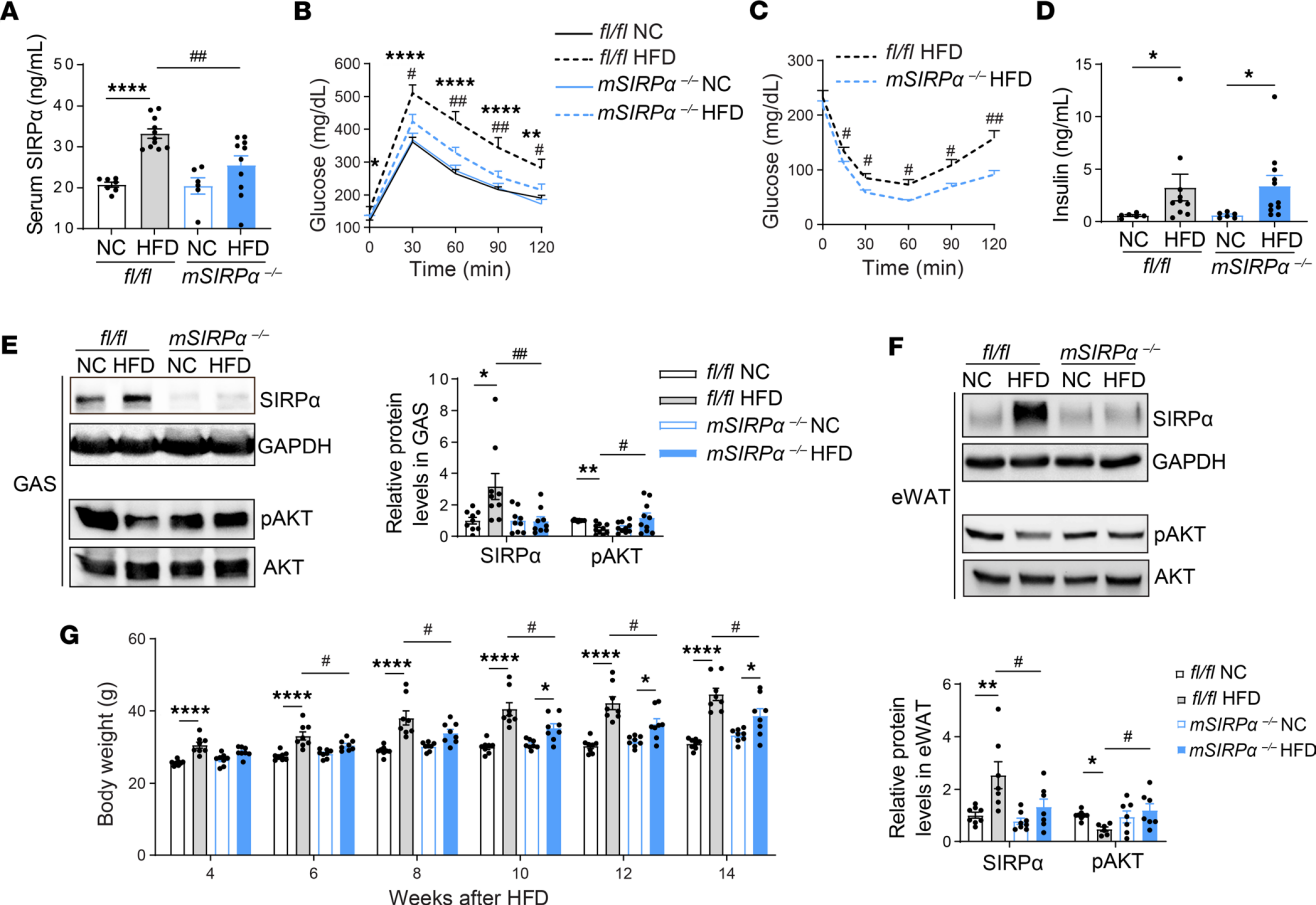
Blocking SIRPα in muscle prevents serum SIRPα release while improving insulin sensitivity in an obesity model of type 2 diabetes. Five-week-old *fl/fl*, muscle-specific SIRPα-KO (*mSIRP*α^–/–^) mice were fed with high-fat diet (HFD) vs. normal chow (NC) diet for 16 weeks. (**A**) Serum SIRPα was confirmed by ELISA (*n* = 6–11). (**B**–**D**) Glucose tolerance (*n* = 7–10), insulin tolerance (*n* = 6–9), and insulin levels (after 16 hours fasting) were measured (*n* = 6–11). (**E** and **F**) Representative immunoblots for SIRPα, pAKT and AKT in gastrocnemius (GAS, *n* = 9-10) skeletal muscle and epididymal white adipose tissue (eWAT, *n* = 6-8) are shown. The relative SIRPα to GAPDH or pAKT to AKT levels are shown. (**G**) Mice body weights (BW) were determined (*n* = 8). Data are shown as mean ± SEM. Statistical significance analysis was performed using 2-tailed unpaired *t* test for **C**; 1-way ANOVA followed by Bonferroni test for **A**, **B**, and **G** and SIRPα in **F**; and Kruskal-Wallis test followed by Dunn’s multiple comparisons for **D** and **E** and pAKT in **F**. *P < 0.05, **P < 0.01, ****P < 0.0001 NC vs. HFD; ^#^*P* < 0.05, ^##^*P* < 0.01 *fl/fl* vs. *mSIRP*α^–/–^.

In order to establish organ-specific SIRPα effects in diabetic disease, muscle-specific SIRPα knockout (KO, ^–/–^) mice were created and fed a 60% high-fat diet (HFD) and compared with normal chow–fed (NC-fed) mice. Serum SIRPα was found to be significantly increased in *flox* mice on HFD, but serum SIRPα was not increased in muscle-specific SIRPα-KO (*mSIRP*α^–/–^) mice on HFD based on ELISA (Figure 1A) and Western blot measurements (Supplemental Figure 1A; supplemental material available online with this article; https://doi.org/10.1172/jci.insight.183392DS1). These results were associated with improved glucose tolerance test (GTT) and insulin tolerance test (ITT) in *mSIRP*α^–/–^ mice on HFD compared with *flox* mice on HFD at 9 weeks and 11 weeks, respectively (Figure 1, B and C), although these differences did not persist beyond 13 weeks (Supplemental Figure 1, B and C), while displaying similar fasting insulin levels in *flox* mice and *mSIRP*α^–/–^ mice on HFD at 14 weeks (Figure 1D).

In gastrocnemius (GAS) skeletal muscle and epididymal white adipose tissue (eWAT), *flox* mice on HFD displayed upregulation of SIRPα proteins with suppressed activation of pAKT (ser 476) signaling (Figure 1, E and F); no differences were noted in intracellular insulin signaling in GAS muscle and eWAT of *mSIRP*α^–/–^ on HFD when compared with their respective control mice on NC while intracellular signaling was improved when compared with *flox* mice on HFD (Figure 1, E and F). Results presented here suggest that the obesity model of type 2 diabetes stimulates increases in serum SIRPα while muscle-specific SIRPα KO leads to improved intracellular insulin signaling in muscle and fat despite HFD feeding. Compared with *flox* mice, obesity was suppressed in *mSIRP*α^–/–^ mice despite HFD beginning at 6 weeks, and these suppressed weight changes persisted until 14 weeks (Figure 1G).

Next, organ weights were evaluated, and *mSIRP*α^–/–^ mice displayed improved inguinal WAT (iWAT), eWAT, and liver weights after HFD feeding when compared with *flox* mice on HFD (Supplemental Figure 1D). These differences were identified despite similar food and water intake (Supplemental Figure 2A). Finally, except for day RER (VCO_2_/VO_2_), no significant differences were identified in VO_2_, VCO_2_, night RER, heat production or activity counts when comparing *flox* vs. *mSIRP*α^–/–^ on HFD (Supplemental Figure 2, B–F). In summary, SIRPα suppression in muscle led to improvements in body and organ weights despite HFD-feed and hyperinsulinemia.

### HFD suppresses FAO genes in adipose tissue while influencing adipokines.

**Figure 2:**
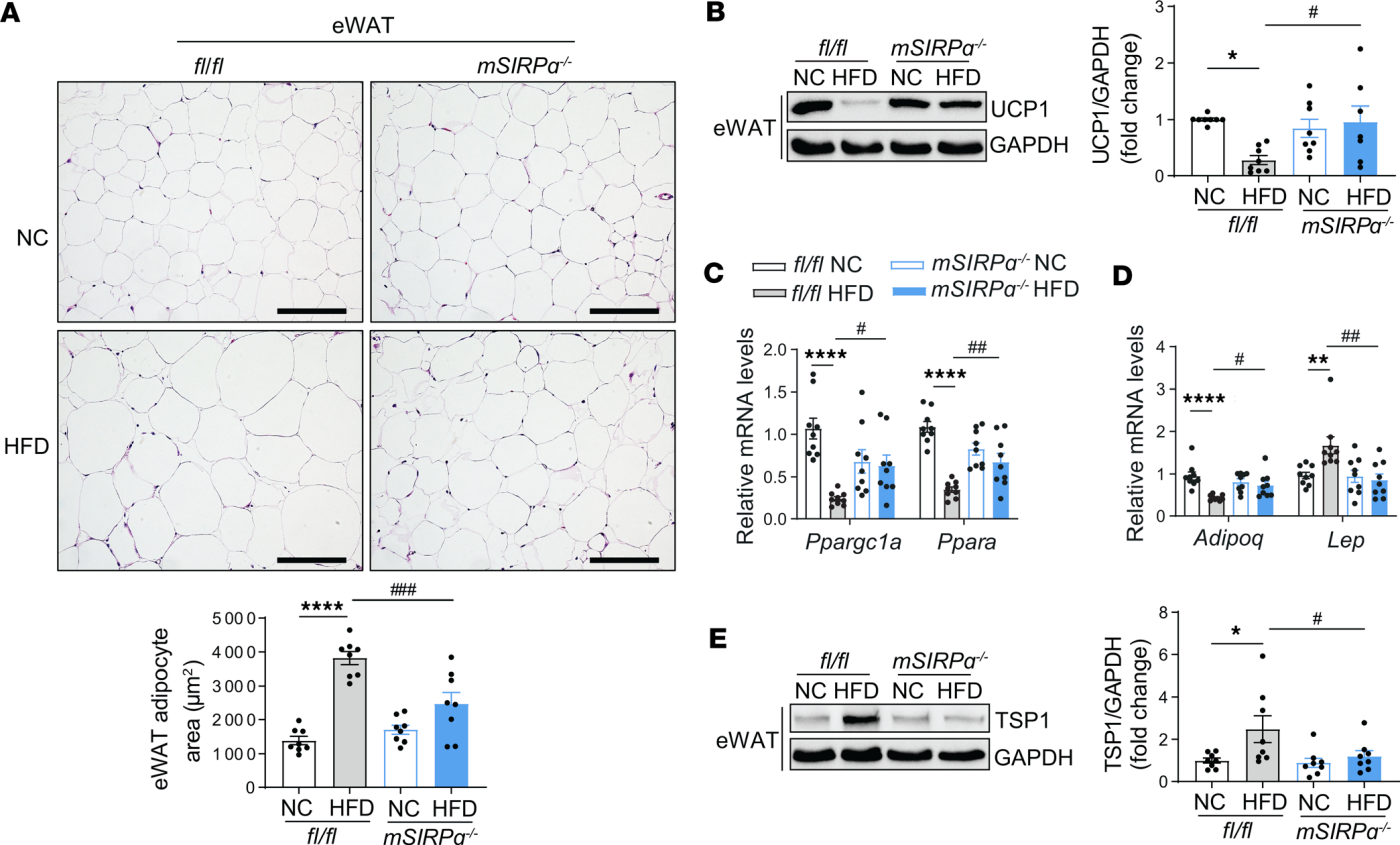
Blocking SIRPα in muscle improves white adipose tissue metabolism. Five-week-old *fl/fl*, muscle-specific SIRPα-KO (*mSIRP*α^–/–^) mice were fed with high-fat diet (HFD) vs. normal chow (NC) diet for 16 weeks. (**A**) Representative H&E-stained epididymal white adipose tissue (eWAT) was obtained and eWAT adipocyte area was measured (scale bar: 100 μm, *n* = 8). (**B**) Representative immunoblots for UCP1 in eWAT and relative densities to GAPDH (*n* = 7–8). (**C**) Relative mRNA fatty oxidation transcript levels were determined in eWAT by qPCR (*n* = 9). (**D**) Relative mRNA levels of adiponectin (*Adipoq*) and leptin (*Lep*) were identified in eWAT by qPCR (*n* = 9). (**E**) Representative immunoblots of adipokine TSP1 in eWAT and the relative densities to GAPDH (*n* = 8). Data are shown as mean ± SEM. Statistical significance was performed using 1-way ANOVA followed by Bonferroni test for **A**–**E**. *P < 0.05, **P < 0.01, ****P < 0.0001 NC vs. HFD; ^#^*P* < 0.05, ^##^*P* < 0.01, ^###^*P* < 0.001 *fl/fl* vs. *mSIRP*α^–/–^.

Next, the effects of SIRPα on eWAT were evaluated in response to obesity-induced diabetes. In *flox* mice, HFD increased eWAT adipocyte area when compared with mice on NC (Figure 2A). However, *mSIRP*α^–/–^ mice on HFD displayed improved adipocyte area similar to *mSIRP*α^–/–^ mice on regular chow (Figure 2A). HFD-fed *flox* mice displayed reduced expression of the thermogenin protein, UCP1, in eWAT when compared with NC-fed *flox* mice, while *mSIRP*α^–/–^ mice on HFD feed showed preservation of UCP1 expression when compared their respective controls (Figure 2B). Additionally, genes *Ppargc1a* encoding PGC1α and *Ppara* encoding PPARα were suppressed in eWAT in response to HFD in *flox* mice but not *mSIRP*α^–/–^ mice on HFD (Figure 2C). Next, we examined adipokine responses; when comparing eWAT of *flox* mice HFD-fed and *flox* NC-fed mice, we observed a reduction in the gene *Adipoq* encoding adiponectin (important for insulin sensitivity) plus an upregulation of *Lep* encoding the satiety hormone leptin (Figure 2D). In contrast, both adiponectin and leptin mRNA levels were unchanged in *mSIRP*α^–/–^ mice on HFD when compared with the NC-fed mice. In fact, *Adipoq* was improved while *Lep* was suppressed in *mSIRP*α^–/–^ mice vs. *flox* mice on HFD (Figure 2D). Similarly, eWAT of HFD-fed *flox* mice displayed increased expression of the adipokine TSP1 (associated with obesity and insulin resistance) but was unchanged in eWAT of HFD-fed *mSIRP*α^–/–^ mice (Figure 2E). Therefore, factors influencing diabetic changes in eWAT were improved in *mSIRP*α^–/–^ mice on HFD.

### SIRPα knockdown improves muscle quality despite hyperglycemia.

**Figure 3:**
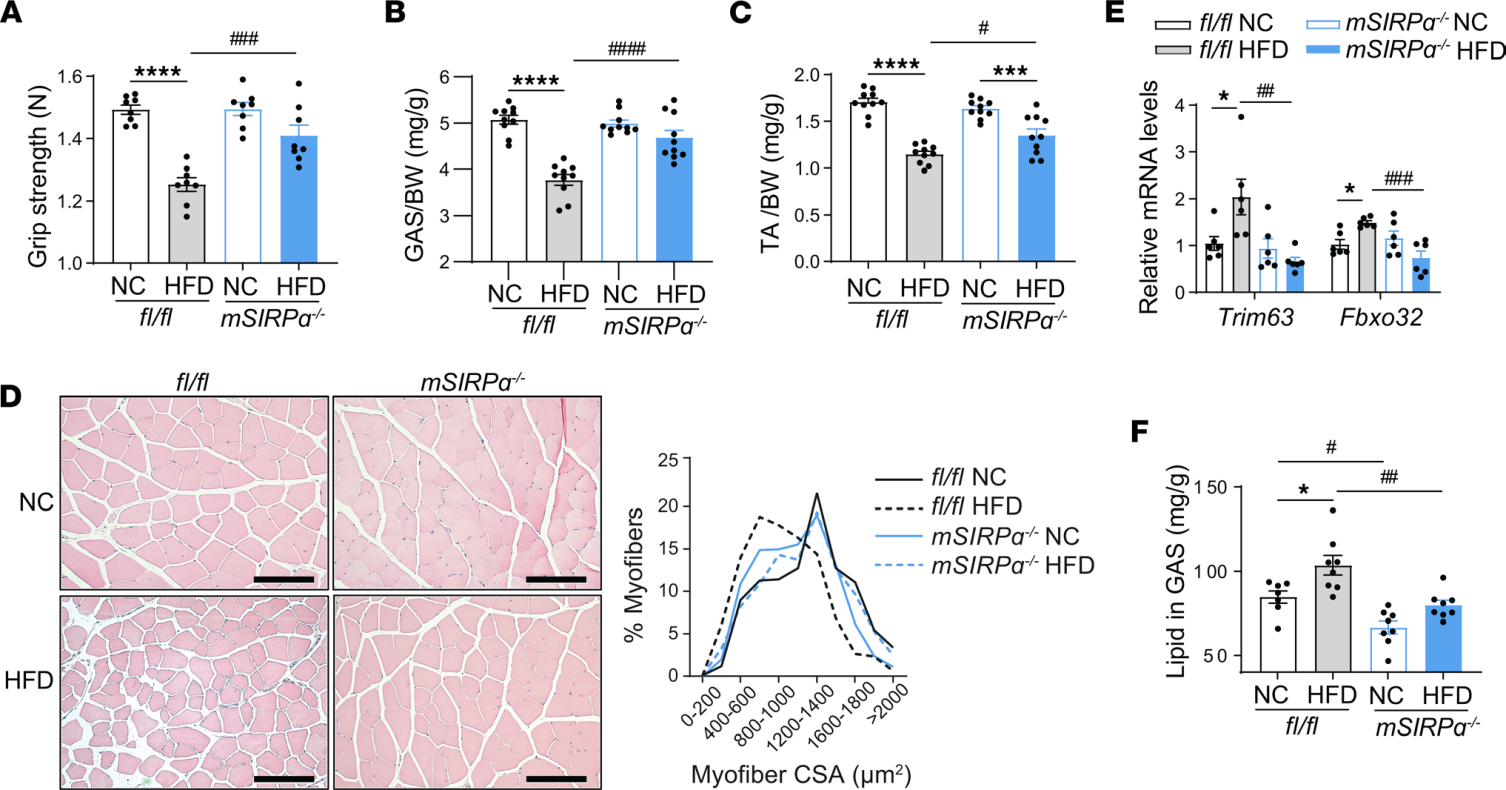
Blocking muscle SIRPα suppresses ectopic lipid accumulation while attenuating skeletal muscle wasting in obesity-induced diabetes. (**A**) After 14 weeks of high-fat diet (HFD) or normal chow (NC) diet, grip strength (Newtons [N]) was measured in *fl/fl*, muscle-specific SIRPα-KO (*mSIRP*α^–/–^) mice (*n* = 8). (**B** and **C**) Gastrocnemius (GAS) and tibialis anterior (TA) skeletal muscles were weighed and normalized to body weight (BW; *n* = 10). (**D**) Representative H&E-stained cryosections of TA skeletal muscle (scale bar: 100 μm) and percentage (%) of myofiber sizes are shown (*n* = 3–4). (**E**) Relative mRNA levels of atrophy-related transcripts were determined in gastrocnemius muscle by qPCR (*n* = 6). (**F**) Total lipid concentration was identified in gastrocnemius skeletal muscle (*n* = 7–8). Data are shown as mean ± SEM. Statistical significance was performed using one-way ANOVA followed by Bonferroni test for **A**–**C**, **E**, and **F**. *P < 0.05, ***P < 0.001, ****P < 0.0001 NC vs. HFD; ^#^*P* < 0.05, ^##^*P* < 0.01, ^###^*P* < 0.001, ^####^*P* < 0.0001 *fl/fl* vs. *mSIRP*α^–/–^.

Skeletal muscle dysfunction is critical to the development of diabetes; therefore, skeletal muscle quality and function were evaluated in response to obesity-induced diabetes. In response to HFD, *flox* mice displayed worsening grip strength and skeletal muscle wasting in both GAS and tibialis anterior (TA) muscle (Figure 3, A–C). However, *mSIRP*α^–/–^ mice vs. *flox* mice on HFD displayed improved grip strength, GAS, and TA skeletal muscle weights (Figure 3, A–C). Cross-sectional areas (CSA) of the TA muscle were reduced in response to HFD in *flox* mice with a leftward shift of CSA but improved in HFD-fed *mSIRP*α^–/–^ with a rightward shift on CSA (Figure 3D). These results were associated with an upregulation in mRNA transcripts responsible for muscle protein degradation E3 ubiquitin ligases, MuRF1 (*Trim63*), and Atrogin-1 (*Fbxo32*) in *flox* mice on HFD with suppression of skeletal muscle E3 ubiquitin ligases in HFD-fed *mSIRP*α^–/–^ (Figure 3E). This is relevant, as these E3 ubiquitin ligase elevations have been associated with muscle protein degradation and atrophy. Of interest, intramuscular lipid was increased in HFD-fed *flox* mice but significantly suppressed in HFD-fed *mSIRP*α^–/–^ (Figure 3F). Thus, blocking muscle SIRPα protected against loss of grip strength and ectopic skeletal muscle lipid deposition, and prevented skeletal muscle wasting despite HFD-feed exposure.

### Obesity-induced diabetes suppresses kidney tubular FAO.

**Figure 4:**
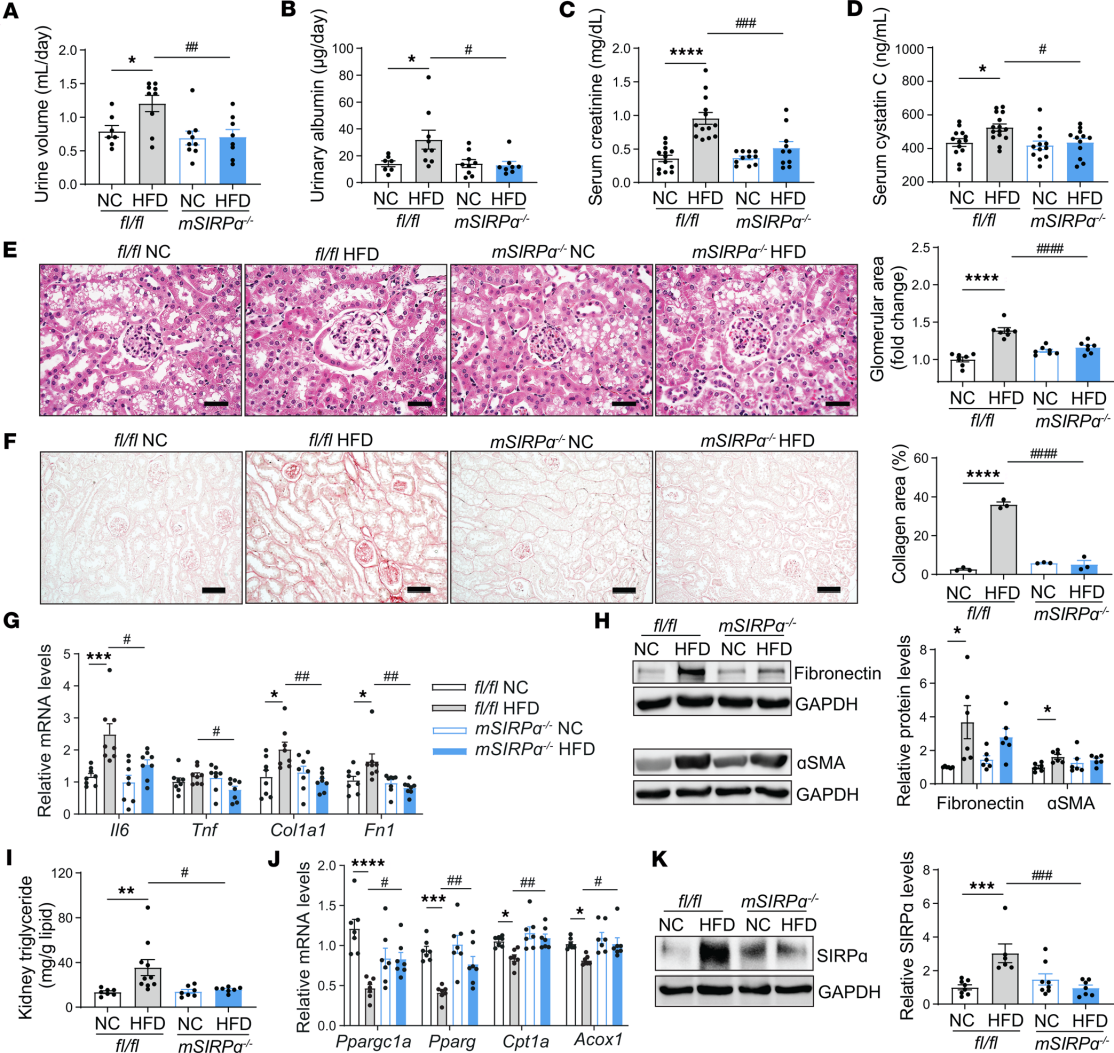
SIRPα suppression in skeletal muscle mitigates HFD-induced renal fibrosis and dysfunction. (**A**–**D**) After 14 weeks on high-fat diet (HFD) vs. normal chow (NC) diet, *fl/fl* and muscle-specific SIRPα-KO (*mSIRP*α^–/–^) mice urine volume (*n* = 7–9), urinary albumin (*n* = 7–9), serum creatinine, and serum cystatin C (*n* = 10–15) were measured. (**E**) Representative H&E-stained kidney glomeruli and quantification of the glomerular size are shown (scale bar: 25 μm, *n* = 7). (**F**) Representative images of sirius red staining of the kidney and percentage (%) area positive for collagen are shown (scale bar: 50 μm, *n* = 3). (**G**) Relative mRNA levels of transcripts related to inflammation and fibrosis were determined by qPCR (*n* = 8). (**H**) Representative immunoblots to fibronectin and α smooth muscle actin (αSMA) and relative densities to GAPDH are identified (*n* = 6). (**I**) Kidney triglyceride to lipid weights were measured (*n* = 7–10). (**J**) Relative mRNA levels of fatty acid oxidation transcripts were determined in the kidney by qPCR (*n* = 7). (**K**) Representative immunoblots of SIRPα in kidney are shown. The relative SIRPα to GAPDH was determined (*n* = 6–8). Data are shown as mean ± SEM. Statistical significance analysis was performed using 1-way ANOVA followed by Bonferroni test for **A**–**K**. *P < 0.05, **P < 0.01, ***P < 0.001, ****P < 0.0001 NC vs. HFD; ^#^*P* < 0.05, ^##^*P* < 0.01, ^###^*P* < 0.001, ^####^*P* < 0.0001 *fl/fl* vs. *mSIRP*α^–/–^.

Therefore, because a reduction in kidney epithelial tubular FAO was noted in patients with hypertensive-diabetic CKD (20), we examined kidney FAO in the HFD model-inducing DKD. Specifically, we evaluated myokine SIRPα effects on kidney epithelial tubular cells. Polyuria, albuminuria, serum creatinine, serum cystatin C, and glomerular area were increased in HFD-fed *flox* mice while suppressed in *mSIRP*α^–/–^mice despite high-fat feeds (Figure 4, A–E). Kidney fibrosis — based on sirius red staining, mRNA transcripts of collagen I (*Col1a1*), fibronectin (*Fn1*), and immunoblots for fibronectin and α smooth muscle actin (αSMA; Figure 4, F–H) — were increased in HFD-fed *flox* mice but not HFD-fed *mSIRP*α^–/–^ mice when compared with their respective controls with improvements in sirius red staining area plus fibrosis-related mRNA transcripts and protein levels in *mSIRP*α^–/–^ mice vs. *flox* mice on HFD (Figure 4, F–H).

Next, glucosuria was increased in HFD-fed *flox* mice but not in *mSIRP*α^–/–^ mice with HFD (Supplemental Figure 3A). No differences were noted in systolic blood pressure (BP) in these mice (Supplemental Figure 3B). Moreover, kidney IL-6 (*Il6*) transcripts were upregulated in HFD-fed *flox* mice vs. control mice, but not in *mSIRP*α^–/–^ mice on HFD, while TNF-α (*Tnf*) was upregulated in *flox* mice on HFD when compared with *mSIRP*α^–/–^ mice on HFD (Figure 4G). Importantly, kidney triglyceride (TG) concentration was found to be significantly increased in HFD-fed *flox* mice, which was significantly reduced in HFD-fed *mSIRP*α^–/–^ mice (Figure 4I). Additionally, genes encoding FAO transcripts (*Ppargc1a*, *Pparg*, *Cpt1a*, *Acox1*) were suppressed in HFD-fed *flox* mice but remained unchanged in *mSIRP*α^–/–^ mice on HFD when compared with their respective controls (Figure 4J). In fact, FAO transcripts were significantly improved in *mSIRP*α^–/–^ mice vs. *flox* mice on HFD (Figure 4J). Finally, SIRPα protein was upregulated in kidneys of *flox* mice on HFD (Figure 4K). In brief, suppressing muscle SIRPα prevented renal fibrosis and changes in fatty acid metabolism while maintaining kidney function in an obesity model of diabetes.

### Exogenous SIRPα suppresses kidney tubular epithelial cells FAO.

**Figure 5:**
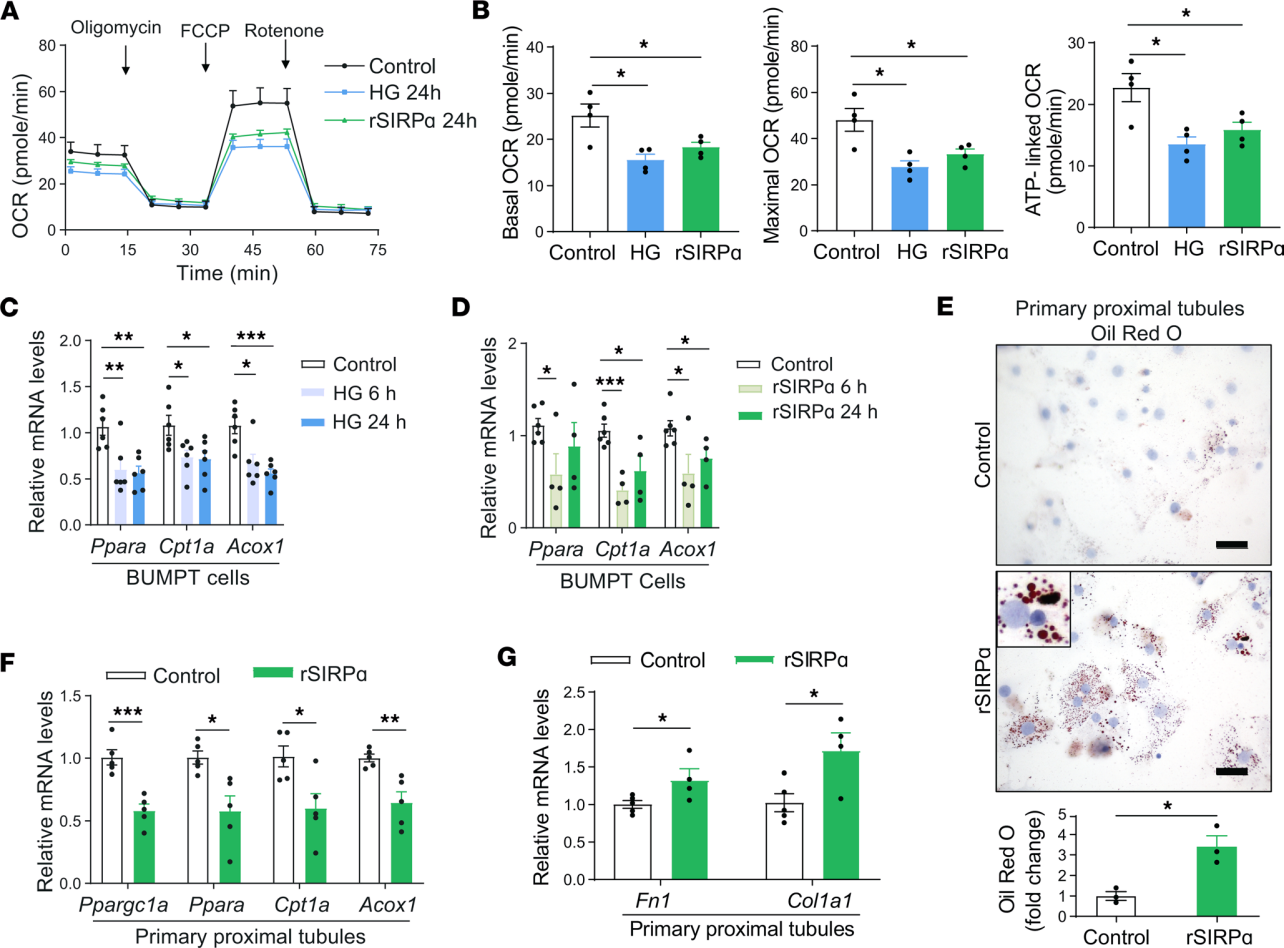
Recombinant SIRPα or hyperglycemia stimulates lipid accumulation in renal proximal tubular cells. (**A** and **B**) Mouse proximal tubular BUMPT cells were incubated with normal glucose (Control, 25 mM) or high glucose (HG, 75 mM) or recombinant SIRPα (rSIRPα) for 24 hours followed by cellular respiration measurements for cellular oxygen consumption rate (OCR) (**A**), basal OCR, maximal OCR, and ATP-linked respiration which were determined by a Seahorse analyzer (**B**) (*n* = 4). (**C**) Relative mRNA levels of fatty acid oxidation transcripts in high glucose–treated BUMPT cells (*n* = 6). (**D**) Relative mRNA levels of fatty acid oxidation transcripts in rSIRPα-treated BUMPT cells (*n* = 4–6). (**E**) Lipid accumulation was determined by Oil red O staining in kidney primary proximal tubules treated with rSIRPα for 24 h (scale bar: 25 μm, *n* = 3). (**F**) Relative mRNA levels of fatty acid oxidation transcripts were determined by qPCR after treatment with rSIRPα in kidney primary proximal tubules (*n* = 5). (**G**) Relative mRNA levels of fibrosis transcripts were determined by qPCR after treatment with rSIRPα in kidney primary proximal tubules (*n* = 4–5). Data are shown as mean ± SEM. Statistical significance was performed using 2-tailed unpaired *t* test for **B**–**G**. *P < 0.05, **P < 0.01, ***P < 0.001 Control vs. HG or rSIRPα-treated groups.

Next, we examined the oxygen consumption rates (OCR) of cultured kidney tubular cells to quantitatively analyze renal proximal tubular metabolism exposed to hyperglycemia or recombinant SIRPα (rSIRPα) protein (Figure 5A). Mouse proximal tubule–derived cell line, BUMPT cells, were treated with a 3-fold higher glucose concentration (75 mM) and compared with standard glucose media (25 mM). High glucose–treated cells had evidence of reduced OCR including basal, maximal, and ATP-linked OCR (Figure 5, A and B). These results were associated with decreased mRNA transcripts encoding FAO (Figure 5C). Next, to determine if exogenous SIRPα directly affects renal proximal tubular FAO, BUMPT cells were exposed to rSIRPα protein. Similar to high glucose–treated cells, rSIRPα-treated cells had evidence of reduced OCR including reduced basal, maximal and ATP-linked OCR similar to high glucose–treated cells (Figure 5, A and B). These outcomes were associated with a reduction in transcript levels for genes encoding FAO in rSIRPα-treated cells (Figure 5D).

Additionally, BUMPT cells were treated at varying concentrations of glucose — 5.5 mM (low) vs. 25 mM (standard) — and OCR were evaluated. Compared with 5.5 mM glucose, 25 mM glucose-treated cells had evidence of reduced OCR including basal, maximal, and ATP-linked OCR (Supplemental Figure 4, A and B). These result findings of 5.5 vs. 25 mM glucose-treated cells were associated with decreased mRNA transcripts encoding FAO (Supplemental Figure 4C).

Next, both primary proximal tubular cells isolated from *flox* control mice kidney cortex (Figure 5, E–G, and Supplemental Figure 4D) and BUMPT cells (Supplemental Figure 4E) were examined after rSIRPα treatment; lipid accumulation was identified based on Oil red O staining at varying time points (Figure 5E and Supplemental Figure 4, D and E) along with downregulation of transcripts encoding genes responsible for FAO (Figure 5F). These results were associated with upregulation of fibronectin (*Fn1*) and collagen I (*Col1a1*) transcripts in primary proximal tubular cells after rSIRPα treatment (Figure 5G). Therefore, exogenous SIRPα reduced both ATP production and FAO transcripts with associated lipid accumulation and stimulation of genes encoding fibrosis in kidney primary proximal tubular cells.

### SIRPα neutralization reverses kidney FAO defects despite streptozotocin-induced hyperglycemia.

**Figure 6:**
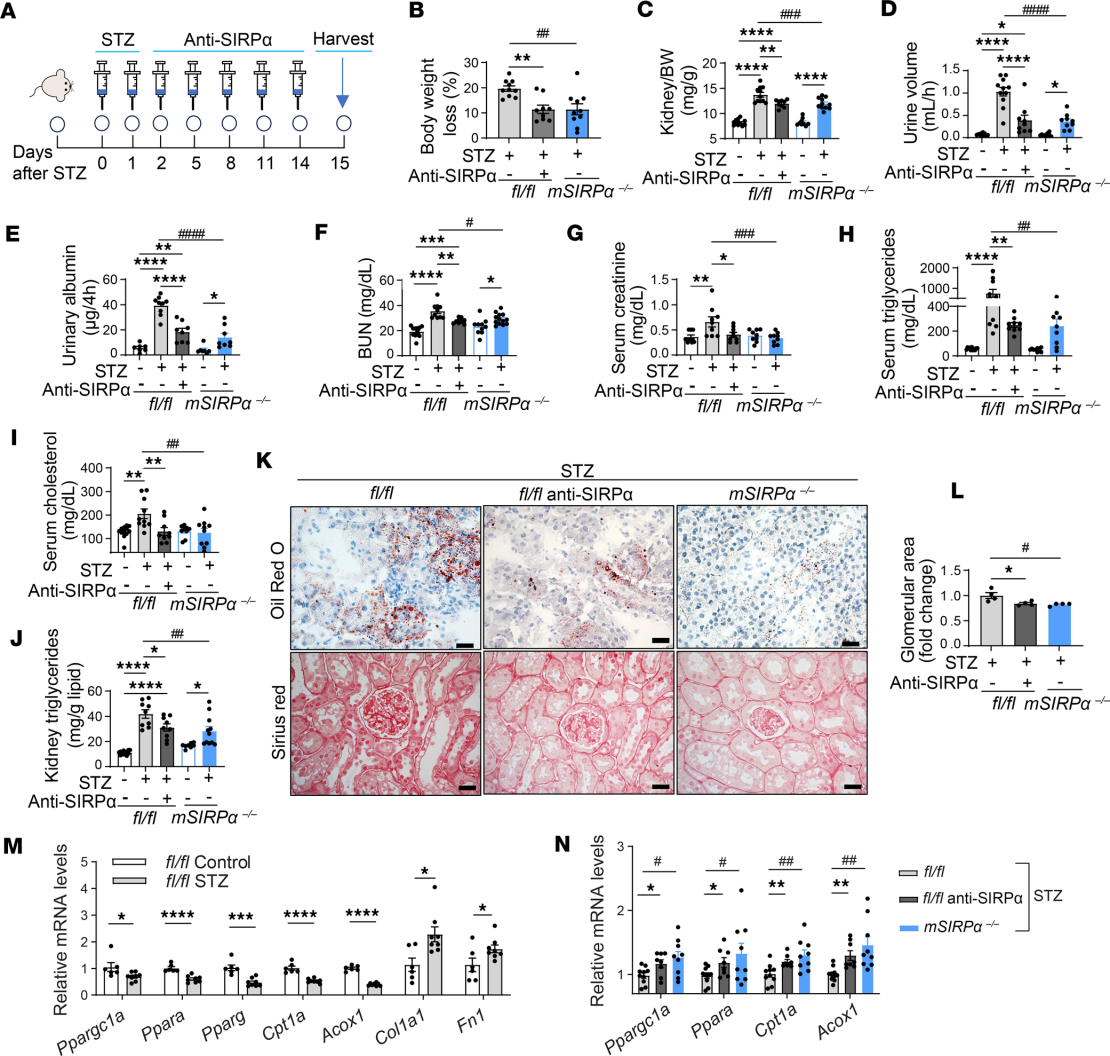
Blocking SIRPα in muscle attenuates streptozotocin-induced cachexia and kidney dysfunction. To induce acute diabetes, streptozotocin (STZ) was injected i.p. into *flox* (*fl/fl*) or muscle-specific SIRPα-KO (*mSIRPα*^–/–^) mice, and measurements listed were made after harvest unless otherwise specified in the methods. (**A**) On day 2 after STZ injection, a group of *fl/fl* mice were given anti-SIRPα monoclonal antibody as illustrated. (**B**–**G**) Next, body weight loss (%), kidney weight, (*n* = 9-12), urine volume, urinary albumin, blood urea nitrogen (BUN), and serum creatinine were measured (*n* = 7–13). (**H** and **I**) Serum triglyceride and total cholesterol were measured (*n* = 9–12) after a 4 hour fast. (**J**) Kidney triglyceride to lipid weight was illustrated (*n* = 8-12). (**K**) Representative images of kidney lipid accumulation based on Oil red O staining and sirius red staining are shown (scale bar: 20 μm). (**L**) Glomerular area is shown (*n* = 4). (**M**) Relative kidney cortex mRNA levels of genes involved in fatty acid oxidation and fibrosis in control vs. STZ-treated *fl/fl* mice (*n* = 6–8). (**N**) Relative mRNA levels of fatty acid oxidation transcripts in kidney cortex were determined by qPCR in STZ-treated *mSIRPα*^–/–^ and *fl/fl* mice ± anti-SIRPα monoclonal antibody treatment (*n* = 8–10). Data are shown as mean ± SEM. Statistical significance was performed using 2-tailed unpaired *t* test for **M** and **N**, and 1-way ANOVA followed by Bonferroni test for the rest. *P < 0.05, **P < 0.01, ***P < 0.001, ****P < 0.0001 Control vs. STZ or *fl/fl* STZ vs *fl/fl* STZ + anti-SIRPα; ^#^*P* < 0.05, ^##^*P* < 0.01, ^###^*P* < 0.001, ^####^*P* < 0.0001 *mSIRPα*^–/–^ STZ vs. *fl/fl* STZ.

To determine insulin-independent effects on kidney FAO, another model of significant hyperglycemia was utilized involving streptozotocin-induced (STZ-induced) insulinopenia. Brouwers et al. determined that STZ-induces worsens kidney function (21). In our study, 3 groups of mice (*fl/fl*, *mSIRP*α^–/–^, and anti-SIRPα monoclonal antibody–treated [mAb–treated] *fl/fl* mice) were treated with STZ (Figure 6A) to induce significant hyperglycemia with blood glucose levels greater than 500 mg/dL. STZ-treated *flox* mice displayed worsening body weight and skeletal muscle losses with increased kidney weights when compared with their respective controls (Figure 6, B and C, and Supplemental Figure 5, A and B), while total body, skeletal muscle, fat, and kidney weights were improved in anti-SIRPα mAb–treated mice or *mSIRP*α^–/–^ mice when compared with *flox* STZ-treated mice (Figure 6, B and C, and Supplemental Figure 5, A–C). Next, polyuria, albuminuria, blood urea nitrogen (BUN), and serum creatinine were increased in *flox* mice treated with STZ, but these parameters were significantly improved in both anti-SIRPα mAb–treated mice or *mSIRP*α^–/–^ mice (Figure 6, D–G). Next, serum TG, total cholesterol (TC), kidney TG, lipid deposition (based on Oil red O staining), and glomerular size were found to be significantly reduced in STZ-treated *mSIRP*α^–/–^ or anti-SIRPα mAb–treated *fl/fl* STZ mice (Figure 6, H–L). However, in *fl/fl* STZ-treated mice, there was evidence of hypercholesterolemia, kidney lipid accumulation, and glomerulomegaly (Figure 6, H–L). Next, SIRPα proteins were found to be reduced in eWAT in *mSIRP*α^–/–^ and anti-SIRPα mAb–treated mice (Supplemental Figure 5D). Additionally, fibronectin proteins were suppressed in *mSIRP*α^–/–^ or anti-SIRPα mAb–treated mice despite STZ when compared with STZ-treated *fl/fl* mice (Supplemental Figure 5E). Additionally, fibrosis was increased based on sirius red and genes encoding fibrosis in STZ-treated *fl/fl* mice but significantly lower when blocking SIRPα in anti-SIRPα mAb–treated mice or *mSIRP*α^–/–^ mice despite STZ (Figure 6, K and M, and Supplemental Figure 5E). Additionally, STZ suppressed renal FAO transcripts in STZ-treated *fl/fl* mice vs. their respective control mice (Figure 6M). In fact, FAO transcripts were found to be improved in *mSIRP*α^–/–^ or anti-SIRPα mAb–treated mice when comparing with *fl/fl* mice after STZ treatment (Figure 6N). In conclusion, these results suggest that blocking SIRPα improved kidney fatty acid metabolism and serum creatinine while preventing features of STZ-induced kidney damage.

### Diabetes stimulates circulating serum SIRPα while exogenous SIRPα suppresses kidney FAO.

**Figure 7:**
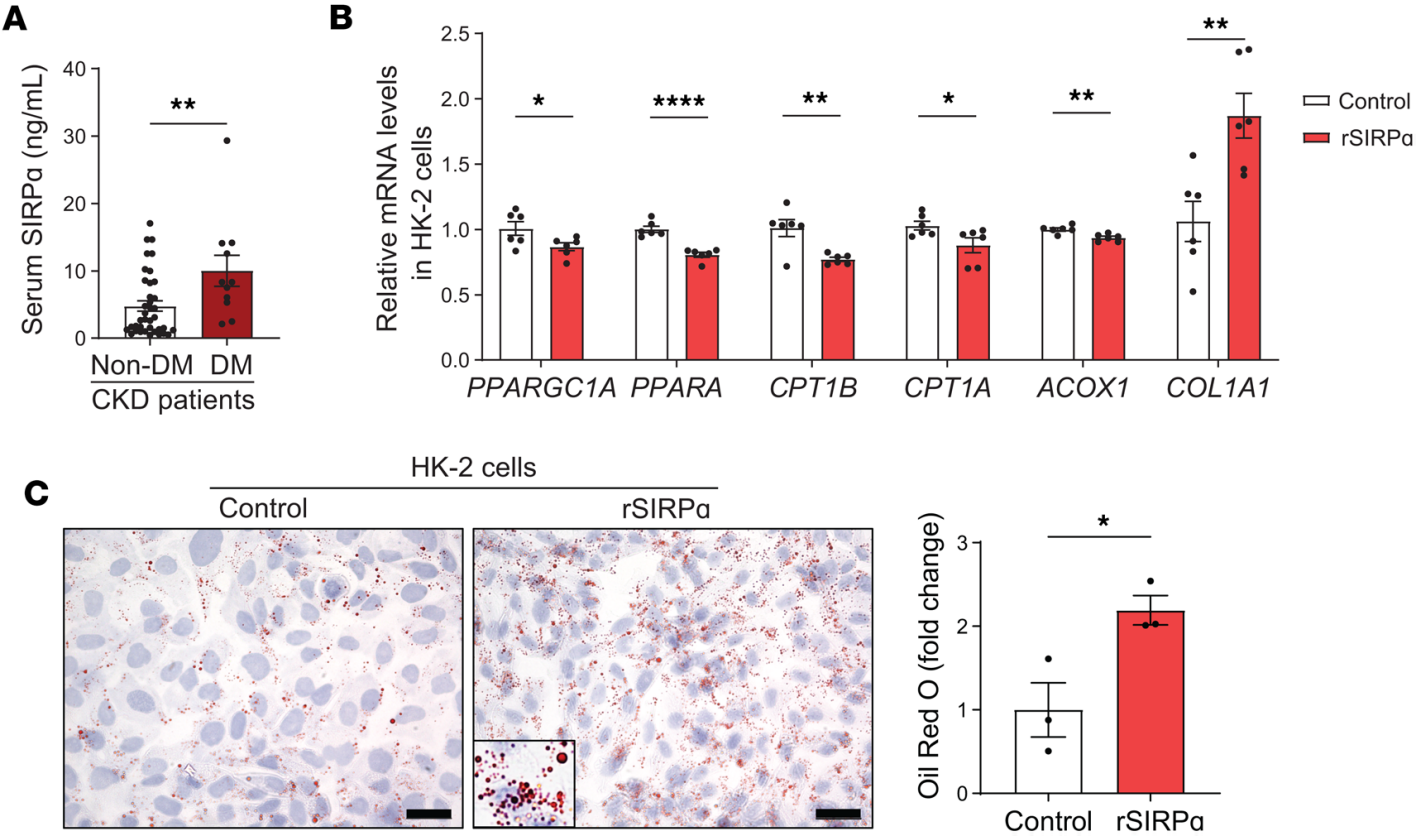
Type 2 diabetes stimulates circulating serum SIRPα in chronic kidney disease. (**A**) Serum SIRPα was measured in patients with type 2 diabetes mellitus (DM, *n* = 11) and non-DM (*n* = 38) patients with chronic kidney disease (CKD). (**B**) Relative mRNA levels of fatty acid oxidation transcripts and fibrosis were determined by qPCR in HK-2 cells exposed to rSIRPα treatment (500 ng/mL) for 24 hours (*n* = 6). (**C**) Lipid accumulation was determined by Oil red O staining in HK-2 cells treated with rSIRPα for 24 hours (scale bar: 25 μm, *n* = 3). Data are shown as mean ± SEM. Statistical significance was performed using 2-tailed unpaired *t* test for **A**–**C**. *P < 0.05, **P < 0.01, ****P < 0.0001.

Finally, we examined serum samples from both diabetic and non-diabetic patients with CKD (Supplemental Table 1). On reanalysis of previous reports (5, 6) plus additional serum samples, patients with diabetes displayed a 2.0-fold significant increase in serum SIRPα (Figure 7A) when compared with nondiabetic CKD serum samples based on ELISA measurements. Therefore, human proximal tubular HK-2 cells were exposed to human rSIRPα; transcripts encoding genes responsible for FAO were suppressed with elevations in type 1 collagen (Figure 7B). Additionally, lipid droplets were increased in those human proximal tubular cells exposed to rSIRPα (Figure 7C). These results suggest that SIRPα may play an important role in human DKD, affecting kidney FAO metabolism in proximal tubules.

## Discussion

This study has noteworthy clinical consequences in identifying the role of myokine SIRPα on kidney function. Muscle secretomes are underinvestigated in renal diseases, specifically the effect of the muscle directly on kidney function and its relevance in organ crosstalk. The mechanisms responsible for disruptions in lipid metabolism in kidney tubular cells in DKD remain unclear. We have determined that CKD stimulates SIRPα to impair insulin signaling in muscle (6). In this study, we examined the effects of myokine SIRPα on kidney disease. Serum SIRPα levels were elevated in patients with CKD (5, 6) and markedly increased in diabetic patients when compared to non-diabetic CKD patients.

Hirai et al. has previously identified that serum SIRPα was cleaved at its extracellular domain (ECD) producing a soluble form after parasitic infection (22). While Vladimirova et al. detected soluble SIRPα in human sera after a lipopolysaccharide (LPS) challenge, and finally Cara-Fuentes et al. detected increases in urinary SIRPα in response to CKD (23, 24). Additionally, we determined that CKD stimulates SIRPα to impair insulin signaling (6). Our current work suggests that muscle-derived SIRPα impairs lipid metabolism and kidney function in the setting of hyperglycemia. Further work is required to identify the biochemical profile including functional role and properties of serum SIRPα.

Two models of hyperglycemia were utilized, an insulin-dependent (HFD) and insulinopenic model (STZ). In our obesity-induced diabetic model, despite evidence of hyperglycemia, suppression of myokine SIRPα led to improved intracellular insulin signaling in peripheral organs with improved body weights in high fat–fed mice regardless of increased insulin levels and hyperglycemia after 13 weeks of exposure. Hyperinsulinemia is associated with weight gain, suppression of lipolysis, while stimulating lipogenesis in adipocytes (25). In fact, intracellular insulin signaling was improved despite hyperinsulinemia and no evidence of glucose lowering effects in *mSIRP*α-KO mice on HFD; therefore, the protective effects were independent of insulin or glucose levels. Improved intracellular insulin skeletal muscle signaling may affect distance organs. Further evaluations are required to determine why the protections were not insulin or glucose dependent. Moreover, thermogenic gene and adipokine expression were improved with a reduction in adipocyte area in *mSIRP*α-KO mice when compared with *flox* mice on HFD, suggesting that fat metabolism was improved. A larger adipocyte area has been reported as an independent predictor of insulin resistance (26).

Additionally, skeletal muscle metabolism was maintained in the presence of hyperglycemia upon skeletal muscle suppression of SIRPα. Skeletal muscles behave as an endocrine organ (27, 28). During exercise, myokines are secreted to communicate with distant organs to enhance fatty acid oxidation and glucose uptake (9, 29). Similar to exercise, suppression of SIRPα improved intramuscular lipid deposition and skeletal muscle losses, however, without any differences noted in oral intake or physical activity.

Lipid droplets, a cellular organelle specialized for storing lipids, are sometimes considered adaptive and cytoprotective against lipotoxicity (30). Although lipids are stored in lipid droplets to ensure homeostasis, ectopic lipid accumulation can be pathognomonic for DKD (31). Kang et al. reported that transgenic mice with CD36 overexpression induces kidney tubular epithelial cell lipid accumulation but that accumulation was not sufficient to induce kidney fibrosis and dysfunction (20).

Next, in control mice exposed to HFD, there is a reduction in fatty acid synthesis transcripts in kidney tissue in mice exposed to HFD (Supplemental Figure 6A) with no changes noted in fatty acid synthesis transcripts in BUMPT cells exposed to rSIRPα treatment (Supplemental Figure 6B). Additionally, rSIRPα treatment of kidney proximal tubular cells did not alter rates of fatty acid uptake (Supplemental Figure 6C). Therefore, reductions in FAO appear to contribute significantly to the dysregulation of metabolism in the obesity-induced model of diabetes. Of note, in spite of hyperglycemia and similar systolic BP, high fat–fed *mSIRP*α-KO mice displayed improved kidney FAO, with a reduction in proteinuria, and improved kidney function (reduced serum creatinine and cystatin C, important biomarkers of DKD; refs. 32, 33). Also, suppressing SIRPα in skeletal muscles or blocking the protein with anti-SIRPα monoclonal antibodies prevented STZ-induced changes in weight loss, glomerulomegaly, and kidney fibrosis, with reduced proteinuria and improved renal FAO and serum creatinine despite evidence of significant hyperglycemia. Additionally, exogenous rSIRPα exacerbated FAO and decreased ATP production, while inducing fibrosis in primary proximal tubular cells similar to hyperglycemia exposure. Additionally, fat droplets were stimulated and FAO suppressed in human proximal tubular cells exposed to rSIRPα treatment. Finally, our in vitro and in vivo data were supported by similar findings in humans, as serum SIRPα was found to be significantly higher in diabetic CKD patients compared with non-diabetic CKD patients. These results suggest a negative effect of myokine SIRPα in kidney disease. Our results are significant, as investigations in renal lipid metabolism are sparse. Specifically, renal TG accumulation has been linked to suppressed FAO expression and kidney fibrosis (20). Kidney function has long been determined based on creatinine, a muscle byproduct (7). Our results highlight the effect of dysregulation of muscle metabolism on kidney function and organ communications. Muscle secretomes including SIRPα are stimulated in response to hyperglycemia or uremic toxins (5).

In models of diabetes with hyperglycemia, suppression of muscle SIRPα improved kidney function. This is the first report to our knowledge to suggest that exogenous SIRPα directly inhibits renal tubular FAO leading to lipid accumulation in proximal tubular epithelial cells with worsening tubulointerstitial fibrosis and kidney dysfunction. The proximal tubular kidney cells share a preference for FAO as an energy source similar to cardiac muscle (6). Prior studies have elucidated the role of increasing lipid accumulation with reduced kidney FAO in DKD (20, 34, 35). These studies did not clarify specific triggers responsible for changes in FAO in renal tubular epithelial cells in DKD. Here we determined that SIRPα independently and directly suppresses renal tubular FAO and ATP production similar to hyperglycemia. In the HFD-induced obesity model, serum SIRPα was found to be increased. Therefore, we conclude that SIRPα exerts effects on DKD to impair renal metabolic homeostasis.

Recent studies have determined that changes in kidney FAO may be a critical factor responsible for kidney dysfunction. For example, signal transducer and activator of transcription 6 (STAT6) activation leads to renal lipid accumulation with suppression of genes responsible for FAO in models of UUO or HFD (36). As well, rho-associated, coiled-coil–containing protein kinase 1 (ROCK1) has been identified a key mediator of FAO in obesity-induced diabetic nephropathy (35). Investigations into metabolic reprogramming or a shift toward a “fasting-like” process improving FA metabolism and/or utilization via SGLT2 inhibitors (SGLT2i) reveal cardio-renal protection (37, 38). Furthermore, SGLT2i may protect proximal tubular metabolic responses in favor of FAO rather than glucose metabolism while inhibiting hypoxia induced factor 1 α (HIF1α) in DKD (38). However, the effect of these studies on muscle-kidney dysfunction in DKD was not investigated. Similarly, the cardiorenal benefit associated with these treatments may be related to changes in FAO metabolism in cardiomyocytes and kidney proximal tubular cells. Future studies are needed to examine cardiac fatty acid metabolism in response to SIRPα suppression in our insulin resistant models; in addition, the timeline of muscle dysfunction and kidney disease requires further evaluation.

In conclusion, changes in muscle metabolism occur decades prior to pancreatic β cell failure or the presentation of diabetes, while exercise proves to reverse the dysregulated metabolic profile (11, 12, 39, 40). However, biomarkers for muscle metabolism are not available when these initial events occur. The importance of our study examines the influence of myokine SIRPα on kidney fatty acid metabolism. CKD is a significant independent risk factor for cardiovascular mortality; further examination is imperative in understanding how dysregulated muscle metabolism impacts kidney function. The relevance of this study details the critical role and effect of muscle on kidney function. Therefore, myokine SIRPα may serve as an important biomarker and therapeutic target in preventing DKD.

## Methods

### Sex as a biological variable.

Our study exclusively examined male mice to limit variability in phenotype. It is unknown whether the findings in mice are relevant to female mice.

### Reagents, chemicals, and antibodies.

The human SIRPα ELISA kit was from LSBio (Seattle, WA). The mouse SIRPα ELISA kit was from LSBio (Shirley, MA). The mouse Cystatin C ELISA kit was obtained from Biovendor R&D (Asheville, NC). The mouse creatinine kit was from Crystal Chem (Elk Grove Village, IL). The cellular fatty acid (C16) uptake assay kit was from Cayman (Ann Arbor, MI). BUN measurements were illustrated by Roman et al. (41). Picrosirius Red Stain Kit was obtained from Polysciences (Warrington, PA). Clarity Urocheck 2 GP Test Strips (Boca Raton, FL) were used for urine glucose measurements. The mouse Albumin ELISA kit was from Crystal Chem (Elk Grove Village, IL). The RNeasy Fibrous Tissue Mini Kit and RNase-Free DNase Set were from Qiagen (Valencia, CA); iScript cDNA Synthesis Kit and iQ SYBR Green Supermix were from Bio-Rad (Hercules, CA). HIScript III RT SuperMix for qPCR (+gDNA wiper) was from Vazyme (Nanjing, China) for the cDNA synthesis of BUMPT cell. The phosphatase inhibitor and protease inhibitor were from Roche (Indianapolis, IN). TRIzol was from Life Technologies (Carlsbad, CA); RIPA lysis and extraction buffer was from G-Biosciences (St. Louis, MO). STZ and insulin were from Sigma Aldrich (St. Louis, MO). The human renal proximal tubular cell line HK-2 from ATCC (Manassas, VA) was cultured in Keratinocyte SFM (1X) media with human recombinant epidermal growth factor (rEGF), bovine pituitary extract (BPE) supplement and fetal bovine serum (FBS) (Gibco, USA). The antibody against GAPDH (Cat. No. MA5-15738) was from Invitrogen (Rockford, IL). SIRPα (Cat. No. 13379), p-AKT (S473, Cat. No. 4060), AKT (Cat. No. 2920), UCP1 (Cat. No. 14670), and TSP1 (Cat. No. 37879) were from Cell Signaling Technology (Beverly, MA). Fibronectin (Cat. No. F3648) and αSMA (Cat. No. A5228) were from Sigma Aldrich (St. Louis, MO). InvivoMAb anti-mouse SIRPα P84 (Cat. No. BE0322) monoclonal antibody was from Bio X Cell (Lebanon, NH).

### Animals.

We studied skeletal muscle-specific SIRPα (*mSIRP*α^–/–^) KO mice or *flox* mice (*SIRPα*^fl/fl^ or *fl/fl*), which were housed in 12-hour light/dark cycles (6 a.m. to 6 p.m.) at 24°C and fed ad libitum either a standard rodent chow or HFD (60%). *SIRPα*^fl/fl^ mice were obtained in conjunction with the BaSH EUCOMM; *mSIRP*α^–/–^ were created with deletion of exons 3 and 4 were generated using Cre (muscle creatine kinase-Cre) mice from The Jackson Laboratory (Bar Harbor, ME) recombinase:loxP system as described previously (6). These genetically modified mice are on *C57BL/6* background with a grossly normal phenotype.

### HFD model.

*SIRPα*^fl/fl^ or *mSIRP*α^–/–^ male mice at 5 weeks of age were randomly allocated to 2 groups: (a) NC with 15% of calories from fat (Cat. No. PicoLab 5V5R, LabDiet, Richmond, IN) or (b) HFD with 60% of calories from fat (Cat. No. D12492, Research Diets, New Brunswick, NJ). The mice were fed with the NC or HFD for 16 weeks. Sixteen-hour fasting serum was used to check serum insulin levels. Random-feeding serum was used to check blood creatinine and cystatin C after 16 weeks of HFD. Urine was collected for 24 hours and used to check albumin and glucose levels after 14 weeks of HFD. For *SIRPα*^fl/fl^ and *mSIRP*α^–/–^ mice, GTT was performed at 9 and 14 weeks; ITT was performed at 11 and 13 weeks using an Advanced Glucose Meter (Woonsocket, RI). For GTT, 16-hour fasted mice were injected i.p. with 1.5 g/kg glucose, and tail vein blood was evaluated at 0-, 30-, 60-, 90-, and 120-minute intervals. For ITT, 5-hour fasted mice were injected i.p. with 1.5 units/kg insulin, and tail vein blood was evaluated at 0-, 15-, 30-, 60-, 90-, and 120-minute intervals to measure blood glucose concentration.

### Whole-body energy metabolism test/ indirect calorimetry.

After 10 weeks on HFD, whole-body energy metabolism of the mice was assessed in the metabolic cages using a Comprehensive Lab Animal Monitoring System (CLAMS, Columbus Instruments, Columbus, OH) in the Mouse Phenotyping Core at BCM. CLAMS cages were housed in temperature-controlled environmental chambers at 23°C on a standard 12-hour light/dark cycles. Animals were monitored for 48 h, and during that time, food and water were provided ad libitum. Parameters monitored include VO_2_, VCO_2_, respiratory exchange ratio (RER), heat, physical activity, and food/water consumption.

### Grip strength.

Forelimb grip strength was assessed after 14 weeks in *SIRPα*^fl/fl^ and *mSIRP*α^–/–^ mice on NC or HFD. Each mouse was pulled backward in a straight, horizontal line to display peak force obtained by Grip Strength Meter (Columbus Instruments, Columbus, OH). Grip strength was measured greater than 3 times for each mouse and the average measurement was illustrated.

### BP measurements.

After 16 weeks of HFD, a noninvasive tail-cuff system (BP-2000, Visitech Systems, Apex, NC) was used to measure indirect BP and heart rate for conscious nonanesthetized mice.

### STZ model.

For acute diabetes, 3-month-old *SIRPα*^fl/fl^ and *mSIRP*α^–/–^ male mice were fasted overnight and administered 150 mg/kg/day 2 days STZ i.p. Food was immediately provided after injections. Mice were monitored for hyperglycemia. The blood glucose reached 373 ± 31 mg/dL 2 days after injection and reached 533 ± 18 mg/dL 1 week after injection and 588 ± 7 mg/dL 2 weeks after STZ infection. On day 2, *SIRPα*^fl/fl^ mice were injected with 200 μg of anti-SIRPα monoclonal antibody or control mice received diluent i.p. every 72 hours for a total of 5 doses before harvest. Mice were fed a NC diet during the experiment. Four-hour fasting serum was used to check BUN, TG, and cholesterol on day 13. Four-hour collection of urine with random feeding was used to check urine volume and albumin on day 14. After harvest on day 15, organ weight, gene transcripts, kidney slides, and serum (filtered by Pierce Spin Columns, Thermo Scientific, Rockford, IL) creatinine were analyzed.

### Proximal tubular cells and in vitro treatment.

Mouse proximal tubular cells were collected from 12-week-old *SIRPα*^fl/fl^ mice fed with NC referred to Peng et al. (9). Mice were euthanized and kidneys were collected in a septic environment. Kidney cortices were dissected visually, minced into small pieces, and digested in 5 mL 37°C preheated DMEM medium containing 2 mg/mL type I collagenase and 1% HEPES (Lonza, Walkersville, MD), then placed into a shaking incubator at 37°C and 5% CO_2_ for 30 minutes. The filtered tissue suspension was collected by a 100 μm strainer (Tisch Scientific, Cleves, OH). Then the samples were centrifuged at 53*g* for 2 minutes to pellet the kidney tubules, washed with DMEM containing 10% FBS and then centrifuged again at 53*g*. The final pellet, consisting mostly of renal tubules, was resuspended in 25 mM glucose DMEM medium supplemented with 10% (v/v) FBS and 1% penicillin-streptomycin (Gibco, USA) and plated in cell culture treated dish and thus these cultured cells were not polarized.

BUMPT cells (the mouse proximal tubule–derived cell line) (in house) were directly seeded in cell culture treated dish with 25 mM glucose DMEM medium containing 10% (v/v) FBS and 1% penicillin-streptomycin at 37°C in 5% CO_2_ to reach 30%–40% confluence by the next day. When the cell confluency reached 80%, they were used for treatment. For high glucose treatment, BUMPT cells were treated with 6 or 24-hour high glucose (75 mM) and compared with standard glucose media (25 mM). For low glucose treatment, BUMPT cells were treated with 6 or 24-hour low glucose (5.5 mM) and compared with standard glucose media (25 mM). BUMPT cells treated with different concentrations of glucose were used to test the cell energy metabolism by the Seahorse XFe96 analyzer (Agilent Technologies, Santa Clara, CA).

Mouse SIRPα recombinant protein (115-125 kDa on SDS-PAGE gel in reducing conditions) was commercially made from the mouse myeloma cell line NS0 (R&D Systems, Minneapolis, MN), specifically not a full-length protein but includes amino acid sequences from Met1-Asn373 in the N-terminus. Human SIRPα recombinant protein (70–105 kDa on SDS-PAGE gel in reducing conditions) was commercially made from Chinese Hamster Ovary (CHO) cell line (R&D Systems, Minneapolis, MN), specifically not a full-length protein but includes amino acid sequences Gly27-Arg370 in the N-terminus. For mouse recombinant SIRPα (rSIRPα) treatment, 500 ng/mL rSIRPα was directly added, without filter, to the media of primary proximal tubular kidney cells or BUMPT cells for 6 h, 24 h, 48 h or 72 h. Cell energy metabolism of BUMPT cells treated with rSIRPα was evaluated by the Seahorse XFe96 analyzer. Fatty acid uptake measurements were made in BUMPT cells treated with or without rSIRPα utilizing Cellular Fatty Acid (C16) Uptake Assay Kit (Cayman, Ann Arbor, MI). Cells were starved for 1 hour in serum-free cultured media and incubated for 1 hour with 500 ng/mL rSIRPα or vehicle control, after which BODIPY-palmitate working solution was added. Negative controls utilized did not contain BODIPY-palmitate. Kinetic measurements of palmitate fatty acid uptake were made every 5 minutes for up to 60 minutes.

Human kidney proximal tubular HK-2 cells from ATCC (Manassas, VA) were cultured in Keratinocyte Serum Free Medium (K-SFM) with supplement of 0.05 mg/mL bovine pituitary extract (BPE), 5 ng/mL human recombinant epidermal growth factor (EGF), 10% FBS and 1% penicillin-streptomycin. HK-2 cells at 80% confluency were exposed to human rSIRPα (500 ng/mL) for 24 h. At the end of incubation, cellular protein was extracted and stored at –80°C.

### Section and staining.

For H&E and sirius red staining, tissue sections (6 μm) were fixed in 4% paraformaldehyde, embedded in paraffin. The myofiber sizes of TA were obtained using NIS-Elements Br 3.0 software (Nikon Instruments, Melville, NY). Frozen cryosections (8 μm) of kidney from STZ mice were mounted on glass slides. For Oil red O (ORO) staining, kidney tissues or cell cultures were fixed in 4% paraformaldehyde for 2 or 30 minutes, respectively, at room temperature, and the slides were stained with a 0.2% Oil red O solution for 15 minutes. Kidney fibrosis was checked by Picrosirius Red Stain Kit. Images were processed using a Nikon 80i microscope (Nikon Instruments, Melville, NY). The experimenter/analyzer was blinded to the treatment groups.

### Western blots.

We homogenized 30 mg kidney/muscle tissue or 100 mg fat tissue for 1 min in 300 μL cold RIPA buffer supplemented with protease and phosphatase inhibitor cocktails. The homogenates were incubated on ice for 10 minutes and then centrifuged (15,600*g*) at 4°C for 15 minutes. The supernatants were used as whole cell lysates. Protein concentration was determined by Pierce BCA Protein Assay (Thermo Scientific, Rockford, IL). An equal amount of protein (80-100 μg) was separated on sodium dodecyl sulfate- polyacrylamide gel electrophoresis in tris/glycine buffer, transferred to nitrocellulose blotting membrane, blocked for 20 minutes, and blotted with all primary antibodies diluted (1:1,000) except fibronectin (1:3,000) in blocking buffer overnight at 4°C. After 3 times of washing in TBS containing 0.1% Tween20, the membrane was incubated in secondary antibody diluted with TBS containing 0.1% Tween20 for 1 hour and washed mentioned earlier above, and the protein was detected using the ChemiDoc MP Imaging System (Bio-Rad, Hercules, CA). The protein of interest was analyzed using Image Lab 6.0 (Bio-Rad, Hercules, CA), and the protein intensities were quantified using NIH ImageJ software. GAPDH was used as an internal standard unless otherwise specified to quantify Western blot bands.

### qPCR.

Total RNA of tissue or cells was extracted using TRIzol. First-strand cDNA was synthesized from 1 μg DNase-treated total RNA using Reverse Transcription Supermix for qPCR. The mRNA levels were evaluated by qPCR using SYBR Green Supermix. Reactions of qPCR were performed in 96-well format using a CFX96 Real-Time System (Bio-Rad, Hercules, CA). The reaction volume was 10 μL, including 5 μL SYBR Green Supermix, 2 μL 2.5 μm primer, and 1 μL cDNA. The following thermal cycling profile was used: 95°C 3 minutes before 40 cycles of 95°C for 15 seconds and 60°C for 30 seconds, followed by 55°C–95°C increment for dissociation curve analysis. The relative mRNA levels were calculated using the comparative 2^–ΔΔCT^ Method (Livak and Schmittgen, 2001) and normalized to *Gapdh* or *Rn18s* (kidney FAO transcripts).

### Primer sequences.

Primer sequences of mouse genes include the following: *Gapdh* F: 5′-TGTGATGGGTGTGAACCACGAGAA-3′, R: 5′-CATGAGCCCTTCCACAATGCCAAA-3′; *Rn18s* F: 5′-GTAACCCGTTGAACCCCATT-3′, R: 5′-CCATCCAATCGGTAGTAGCG-3′; *Ppargc1a* F: 5′-TCCTCTTCAAGATCCTGTTAC-3′, R: 5′-CACATACAAGGGAGAATTGC-3′; *Ppara* F: 5′-GAATCCACGAAGCCTACC-3′, R: 5′-GCCATACACAAGGTCTCC-3′; *Pparg* F: 5′-GCGGTGAACCACTGATAT-3′, R: 5′-TGGCATCTCTGTGTCAAC-3′; *Acox1* F: 5′-CAGAGTTAATCACGCACATC-3′, R: 5′-TGGATCGTTCAGAATCAAGT-3′; *Cpt1a* F: 5′-GGTCTTCTCGGGTCGAAAGC-3′, R: 5′-TCCTCCCACCAGTCACTCAC-3′; *Fasn* F: 5′-GAGCCCAGACAGAGAAGA-3′, R: 5′-GTCCACACCACCAATGAG-3′; *Acaca* F: 5′-ATTGGGCACCCCAGAGCTA-3′, R: 5′-CCCGCTCCTTCAACTTGCT-3′; *Srebf1* F: 5′-AATAAATCTGCTGTCTTGCG-3′, R: 5′-CCTTCAGTGATTTGCTTTTG-3′; *Il6* F: 5′-AGCCAGAGTCCTTCAGAGAGA-3′, R: 5′-TAGGAGAGCATTGGAAATTGGG-3′; *Tnf* F: 5′-CATGAGCACAGAAAGCATGATCCG-3′, R: 5′-AAGCAGGAATGAGAAGAGGCTGAG-3′; *Trim63* F: 5′-GTGTGAGGTGCCTACTTGCTC-3′, R: 5′-GCTCAGTCTTCTGTCCTTGGA-3′; *Fbxo32* F: 5′-CTGAAAGTTCTTGAAGACCAG-3′, R: 5′-GTGTGCATAAGGATGTGTAG-3′; *Fn1* F: 5′-AATCGTGCAGCCTCAATC-3′, R: 5′-CCTCCATAGCAGGTACAAAC-3′; *Col1a1* F: 5′-GTTCAGCTTTGTGGACCTC-3′, R: 5′-GGCAGATACAGATCAAGCAT-3′; *Lep* F: 5′-CTTTGGTCCTATCTGTCTTATG-3′, R: 5′-TCTTGGACAAACTCAGAATG-3′; *Adipoq* F: 5′-CCACTTTCTCCTCATTTCTG-3′, R: 5′-CTAGCTCTTCAGTTGTAGTAAC-3′.

### Human genes.

Primer sequences of human genes include the following: *GAPDH* F: 5′-CTTTTGCGTCGCCAG-3′, R: 5′-TTGATGGCAACAATATCCAC-3′; *PPARGC1A* F: 5′-GCAGACCTAGATTCAAACTC-3′, R: 5′-CATCCCTCTGTCATCCTC-3′; *PPARA* F: 5′-CCTAAAAAGCCTAAGGAAACC-3′, R: 5′-GATCTCCACAGCAAATGATAG-3′; *CPT1A* F: 5′-ACGGGGATTATAAGTCAAGG-3′, R: 5′-CACAGCAAGTGAAAATCAAC-3′; *CPT1B* F: 5′-AGAATTCCAGGACAAGACTG-3′, R: 5′-CACTCACATAGTTACTTGCC-3′; *ACOX1* F: 5′-AAAGCAGAGGTCCACG-3′, R: 5′-CCACAAAATTTGAGTTGCAC-3′; *COL1A1* F: GCTATGATGAGAAATCAACCG-3′, R: 5′-TCATCTCCATTCTTTCCAGG-3′.

### Statistics.

Values are expressed as mean ± SEM. Significance analysis was performed using 2-tailed unpaired Student’s *t* test to compare data between 2 groups and 1-way ANOVA followed by Bonferroni for multiple variables unless otherwise specified in the figure legends. *P* < 0.05 was considered statistically significant. GraphPad Prism software was used for statistical analysis.

### Study approval.

All animal procedures were performed in accordance with the standards set forth in the guidelines for IACUC of BCM.

The procedures for human samples were approved by the Ethics Committee of the Department of Internal Medicine of the University of Genoa, in accordance with the Declaration of Helsinki regarding ethics of human research. Serum samples were obtained from patients with advanced kidney disease. Before the patients’ participation, the nature, purpose, and risks of the study were reviewed with all the participants, and their voluntary consent was obtained. The detailed characteristics of patients with CKD are available in Supplemental Table 1.

### Data availability.

All data associated with this study are present in the paper or the supplemental information, and raw data are included in the Supporting Data Values file.

## Author contributions

SST and JW carried out experiments, study design, figure legends, and data analysis. DSH, QL, and ZH contributed to experiments related to the Seahorse experiment. ER and DV contributed to sample collection and measurements. JW, BK, and HZ contributed to histological examination, while WEM contributed to editing. SST, the principal investigator, conceptualized the study, obtained funding, and drafted the manuscript. All the authors reviewed, contributed to, and approved the final manuscript.

## Funding support

- VA Merit Award 1I01BX005792 to SST from the United States Department of Veterans Affairs, Biomedical Laboratory Research and Development Program.
- 2I01BX002006 to DSH from the United States Department of Veterans Affairs, Biomedical Laboratory Research and Development Program.
- American Society of Nephrology Carl W. Gottschalk Research Scholar Grant to SST.
- Baylor College of Medicine MMPC, funded by NIH RO1DK114356 & UM1HG006348.

## Supplementary Material

Supplemental data

Unedited blot and gel images

Supporting data values

## Version 1 02/09/2026

Electronic publication

## References

[1] Gonzalez-Rocha A (2023). Risk prediction score for chronic kidney disease in healthy adults and adults with type 2 diabetes: systematic review. Prev Chronic Dis.

[2] Koye DN (2018). The global epidemiology of diabetes and kidney disease. Adv Chronic Kidney Dis.

[3] Fliser D (1998). Insulin resistance and hyperinsulinemia are already present in patients with incipient renal disease. Kidney Int.

[4] Thomas SS (2013). Signal regulatory protein-α interacts with the insulin receptor contributing to muscle wasting in chronic kidney disease. Kidney Int.

[5] Thomas SS (2022). SIRPα mediates IGF1 receptor in cardiomyopathy induced by chronic kidney disease. Circ Res.

[6] Wu J (2019). Signal regulatory protein alpha initiates cachexia through muscle to adipose tissue crosstalk. J Cachexia Sarcopenia Muscle.

[7] Patel SS (2013). Serum creatinine as a marker of muscle mass in chronic kidney disease: results of a cross-sectional study and review of literature. J Cachexia Sarcopenia Muscle.

[8] Verzola D (2020). Enhanced myostatin expression and signalling promote tubulointerstitial inflammation in diabetic nephropathy. Sci Rep.

[9] Peng H (2017). Myokine mediated muscle-kidney crosstalk suppresses metabolic reprogramming and fibrosis in damaged kidneys. Nat Commun.

[10] Hanatani S (2014). Akt1-mediated fast/glycolytic skeletal muscle growth attenuates renal damage in experimental kidney disease. J Am Soc Nephrol.

[11] Kosmadakis GC (2012). Benefits of regular walking exercise in advanced pre-dialysis chronic kidney disease. Nephrol Dial Transplant.

[12] Mustata S (2011). Effects of exercise training on physical impairment, arterial stiffness and health-related quality of life in patients with chronic kidney disease: a pilot study. Int Urol Nephrol.

[13] Traise A (2024). The effect of exercise training in people with pre-dialysis chronic kidney disease: a systematic review with meta-analysis. J Nephrol.

[14] Shurraw S (2011). Association between glycemic control and adverse outcomes in people with diabetes mellitus and chronic kidney disease: a population-based cohort study. Arch Intern Med.

[15] Action to Control Cardiovascular Risk in Diabetes Study Group (2008). Effects of intensive glucose lowering in type 2 diabetes. N Engl J Med.

[16] Afshinnia F (2019). Increased lipogenesis and impaired β-oxidation predict type 2 diabetic kidney disease progression in American Indians. JCI Insight.

[17] Bhargava P, Schnellmann RG (2017). Mitochondrial energetics in the kidney. Nat Rev Nephrol.

[18] Pagliarini DJ (2008). A mitochondrial protein compendium elucidates complex I disease biology. Cell.

[19] Rinaldi A (2022). Impaired fatty acid metabolism perpetuates lipotoxicity along the transition to chronic kidney injury. JCI Insight.

[20] Kang HM (2015). Defective fatty acid oxidation in renal tubular epithelial cells has a key role in kidney fibrosis development. Nat Med.

[21] Brouwers B (2013). Phlorizin pretreatment reduces acute renal toxicity in a mouse model for diabetic nephropathy. J Biol Chem.

[22] Hirai H (2023). Leishmania infection-induced proteolytic processing of SIRPα in macrophages. Pathogens.

[23] Vladimirova YV (2022). A new serum macrophage checkpoint biomarker for innate immunotherapy: soluble signal-regulatory protein alpha (sSIRPα). Biomolecules.

[24] Cara-Fuentes G (2022). Pulmonary surfactants and the respiratory-renal connection in steroid-sensitive nephrotic syndrome of childhood. iScience.

[25] Kolb H (2018). Insulin translates unfavourable lifestyle into obesity. BMC Med.

[26] Kloting N (2010). Insulin-sensitive obesity. Am J Physiol Endocrinol Metab.

[27] Severinsen MCK, Pedersen BK (2020). Muscle-organ crosstalk: the emerging roles of myokines. Endocr Rev.

[28] Ma S (2023). Skeletal muscle-derived extracellular vesicles transport glycolytic enzymes to mediate muscle-to-bone crosstalk. Cell Metab.

[29] Baar K (2002). Adaptations of skeletal muscle to exercise: rapid increase in the transcriptional coactivator PGC-1. FASEB J.

[30] Pressly JD (2022). Adaptive and maladaptive roles of lipid droplets in health and disease. Am J Physiol Cell Physiol.

[31] Mitrofanova A (2023). Kidney lipid dysmetabolism and lipid droplet accumulation in chronic kidney disease. Nat Rev Nephrol.

[32] Mussap M (2002). Cystatin C is a more sensitive marker than creatinine for the estimation of GFR in type 2 diabetic patients. Kidney Int.

[33] Sapkota S (2021). Diagnostic accuracy of serum cystatin C for early recognition of nephropathy in type 2 diabetes mellitus. Int J Nephrol.

[34] Herman-Edelstein M (2014). Altered renal lipid metabolism and renal lipid accumulation in human diabetic nephropathy. J Lipid Res.

[35] Nagai Y (2022). Rho-associated, coiled-coil-containing protein kinase 1 regulates development of diabetic kidney disease via modulation of fatty acid metabolism. Kidney Int.

[36] Li J (2022). STAT6 contributes to renal fibrosis by modulating PPARα-mediated tubular fatty acid oxidation. Cell Death Dis.

[37] Gao YM (2022). Cardiorenal protection of SGLT2 inhibitors-perspectives from metabolic reprogramming. EBioMedicine.

[38] Cai T (2020). Sodium-glucose cotransporter 2 inhibition suppresses HIF-1α-mediated metabolic switch from lipid oxidation to glycolysis in kidney tubule cells of diabetic mice. Cell Death Dis.

[39] Bishop NC (2023). Exercise and chronic kidney disease: potential mechanisms underlying the physiological benefits. Nat Rev Nephrol.

[40] DeFronzo RA, Tripathy D (2009). Skeletal muscle insulin resistance is the primary defect in type 2 diabetes. Diabetes Care.

[41] Roman RJ (1979). Fluorometric assay for urea in urine, plasma, and tubular fluid. Anal Biochem.

