# Supplementary material for: Myokine SIRPα exacerbates kidney disease in diabetes
Source: JCI Insight. 2026 Feb 9;11(3):e183392. doi: 10.1172/jci.insight.183392 (PMC12893106; doi:10.1172/jci.insight.183392)
Supplement: Supplemental data [file jciinsight-11-183392-s290.pdf]

Supplementary Figures

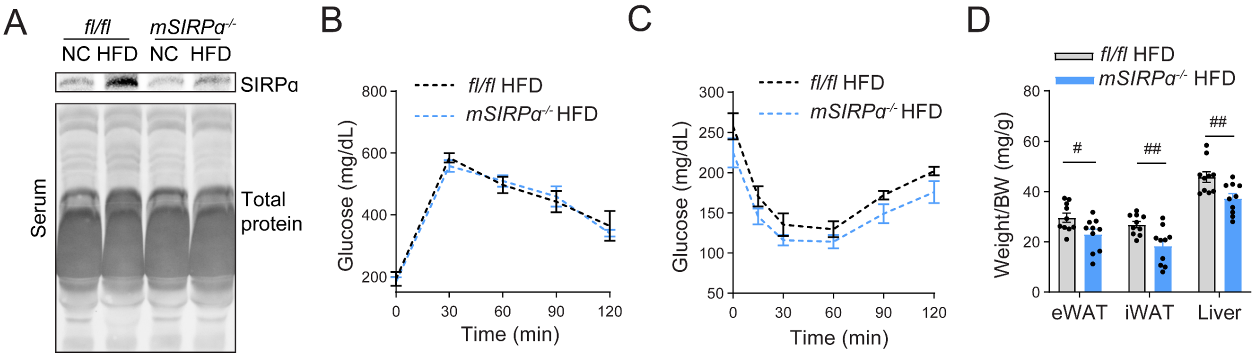

Figure S1. SIRPα suppression in muscle reduces HFD-induced fat accumulation and hepatic weight. *Flox* (*fl/fl*) and muscle-specific SIRPα knockout (KO; *mSIRPα*$^{-/-}$) mice were treated with high fat diet (HFD) vs. normal chow (NC) diet for 16 weeks. (**A**) Representative immunoblots for serum SIRPα are shown. After 13 weeks on HFD, glucose tolerance (**B**, n=6) and insulin tolerance tests (**C**, n=5-6) were performed in *fl/fl* and *mSIRPα*$^{-/-}$ mice. (**D**) Epididymal white adipose tissue (eWAT), inguinal white adipose tissue (iWAT) and liver were measured and normalized to body weight (BW) in *fl/fl* and *mSIRPα*$^{-/-}$ mice after HFD (n=10). Values are expressed as a mean ± SEM. Statistical significance was performed using two-tailed unpaired t-test for **B**-**D**. #P < 0.05, ##P < 0.01 *fl/fl* vs. *mSIRPα*$^{-/-}$ on HFD.

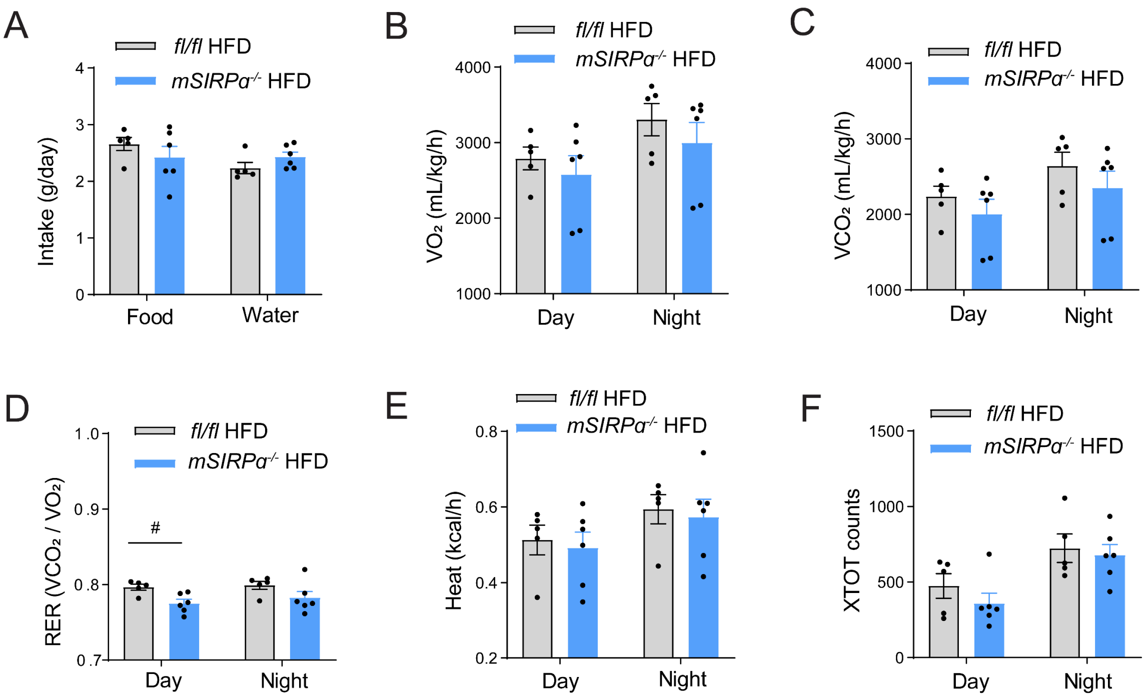

Figure S2. Blocking SIRPα does not impact heat generation or energy expenditure in obesity-induced diabetes. Five-week-old *fl/fl* mice vs. muscle-specific SIRPα knockout (KO; *mSIRPα*$^{-/-}$) mice were treated with high fat diet (HFD) for 10 weeks. (**A**)Measurements of food and water intake in 48 h, (**B-F**) VO2, VCO2, respiratory exchange ratio (RER), heat and physical activity were monitored by indirect calorimetry (n=5-6). Values are expressed as a mean ± SEM. Statistical significance was performed using two-tailed unpaired t-test for **A-F**. #P < 0.05 *fl/fl* vs. *mSIRPα*$^{-/-}$ mice on HFD.

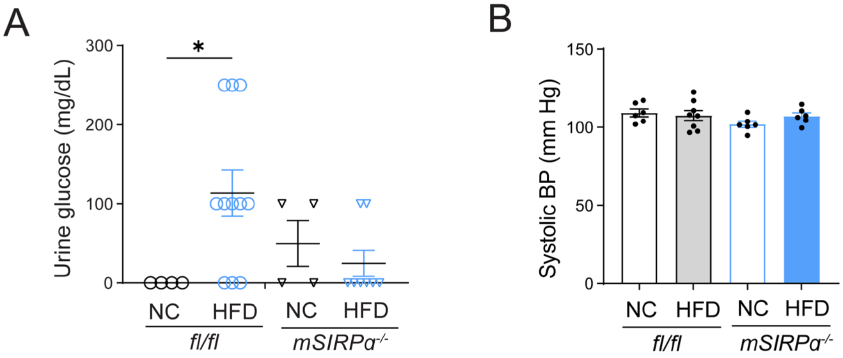

Figure S3. Metabolic parameters after high fat diet. Five-week-old *fl/fl* mice vs. muscle-specific SIRPα knockout (KO; *mSIRPα*$^{-/-}$) were treated with normal chow (NC) or high fat diet (HFD). (**A**) Urine glucose was measured after 14 weeks of HFD (n=4-11). (**B**) Systolic blood pressure (BP) was measured after 16 weeks of HFD (n=6-8). Values are expressed as a mean ± SEM. Kruskal-Wallis test followed by Dunn's multiple comparisons for **A**; one-way ANOVA followed by Bonferroni test for **B**. *P < 0.05 *fl/fl* mice on NC vs. HFD.

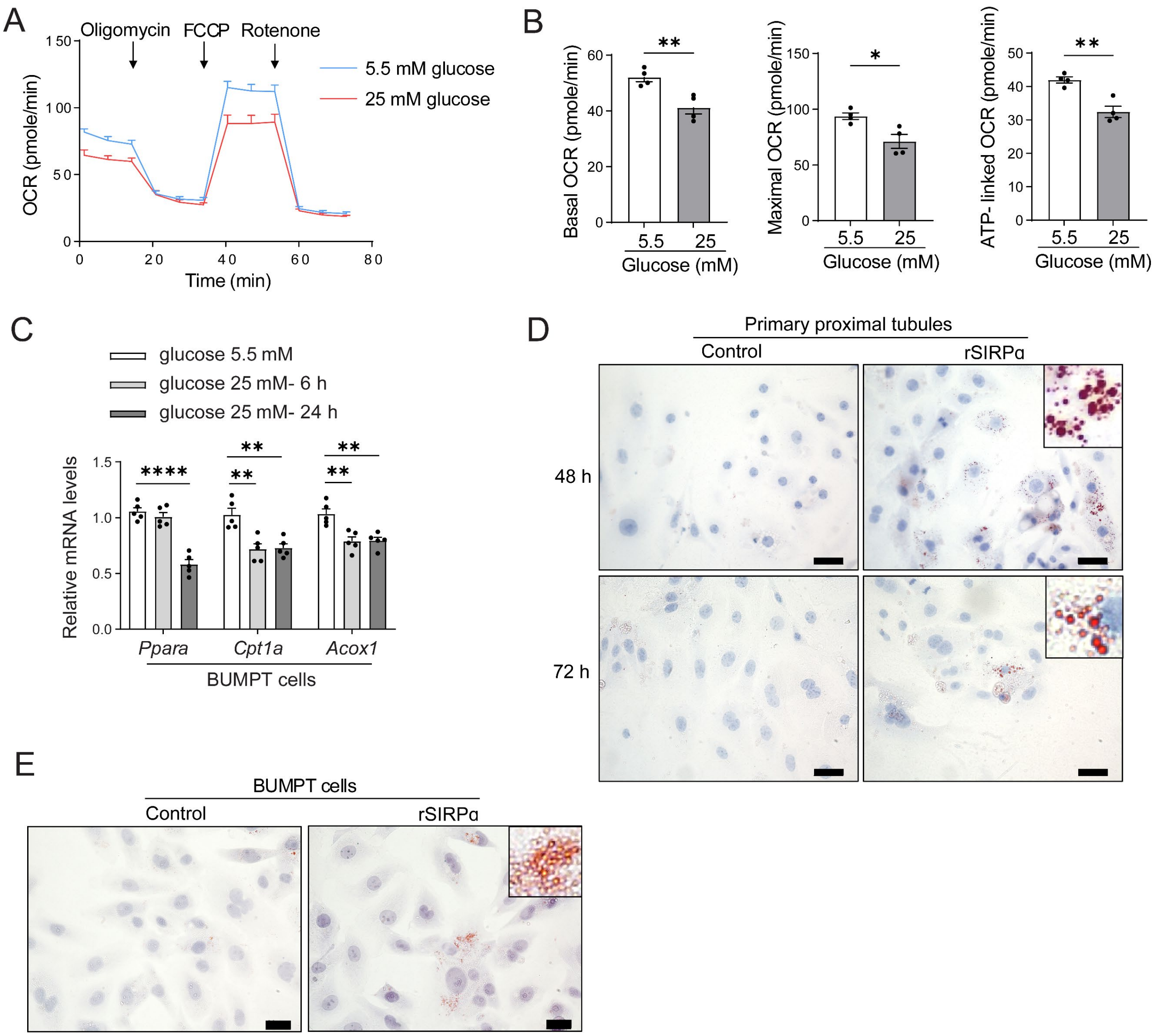

Figure S4. Recombinant SIRPα (rSIRPα) stimulates lipid accumulation in renal proximal tubular cells. Mouse proximal tubular BUMPT cells were incubated with low glucose (5.5 mM) or normal glucose (25 mM) for 24 hours followed by cellular respiration measurements for (**A**) cellular oxygen consumption rate (OCR); (**B**) basal OCR, maximum OCR, and ATP-linked OCR which were determined by a Seahorse analyzer (n= 4) and (**C**) relative mRNA levels of fatty acid oxidation transcripts (n=5). (**D**) Representative images of Oil Red O staining of kidney primary proximal tubules incubated with rSIRPα for 48 or 72 h (scale bars=25 μm). (**E**) Representative images of Oil Red O staining of BUMPT cells treated with rSIRPα for 24 h (scale bars=20 μm). Values are expressed as a mean ± SEM. Statistical significance was performed using two-tailed unpaired t-test for **B** and **C**. *P < 0.05, **P < 0.01, ****P <0.0001 5.5 mM vs 25 mM glucose.

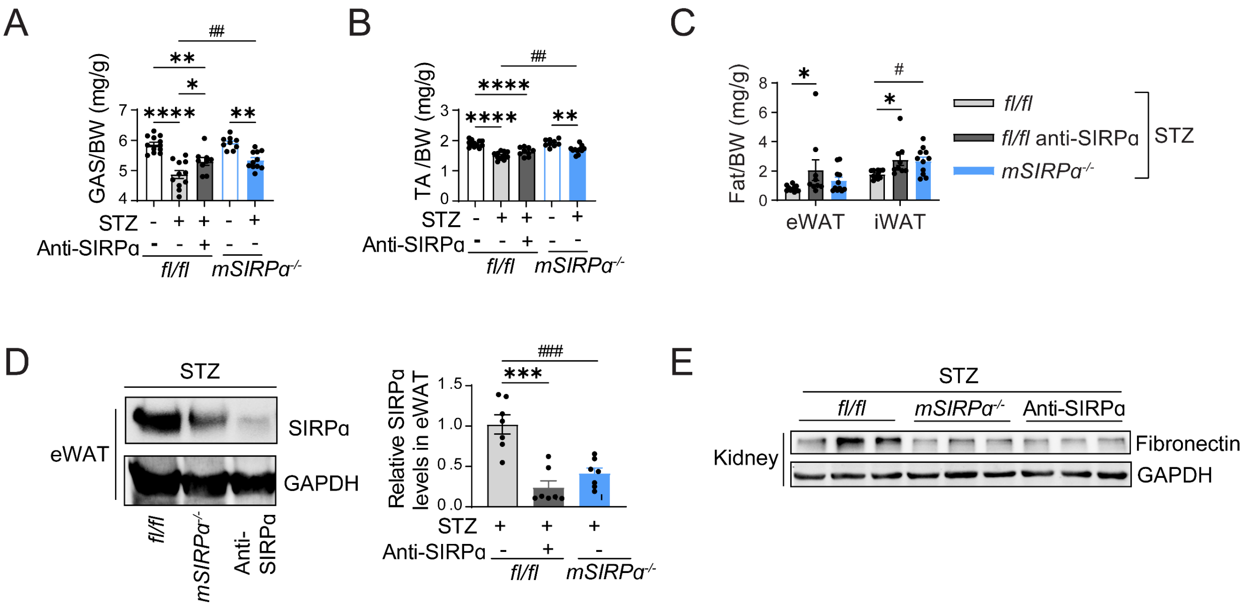

Figure S5. Blocking SIRPα prevents streptozotocin (STZ)-induced metabolic changes. Skeletal muscles including (**A**) gastrocnemius (GAS) and (**B**) tibialis anterior (TA) were weighed and normalized to body weight (BW). (**C**) Weight of epididymal white adipose tissue (eWAT) and inguinal white adipose tissue (iWAT) were normalized to BW (n=9-12) after STZ treatment. (**D**) Representative immunoblots for SIRPα in epididymal white adipose tissue (eWAT) after STZ in *fl/fl*, *mSIRPα*$^{-/-}$ or anti-SIRPα mAb-treated mice with quantification relative to GAPDH (n=7). (**E**) Representative immunoblots for fibronectin in kidney cortex after STZ treatment in *fl/fl*, *mSIRPα*$^{-/-}$ or anti-SIRPα mAb-treated mice. Values are expressed as a mean ± SEM. Statistical significance analysis was performed using one-way ANOVA followed by Bonferroni test for **A**-**C** and two-tailed unpaired t-test for **D.** $^*P < 0.05$, $^{**}P < 0.01$, $^{***}P<0.001$, $^{****}P<0.0001$ Control vs. STZ or *fl/fl* STZ vs. *fl/fl* STZ + anti-SIRPα mAb; #P < 0.05, ##P < 0.01, ###P < 0.001 *fl/fl* STZ vs. *mSIRPα*$^{-/-}$ STZ.

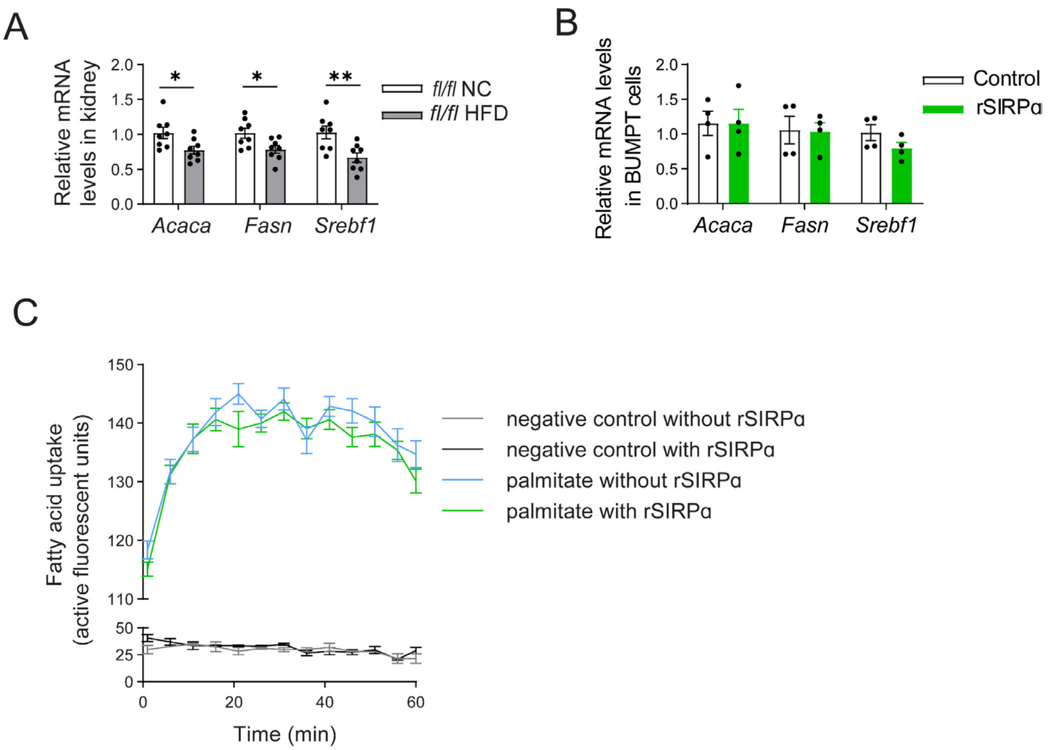

Figure S6. Fatty acid synthesis and uptake effects of SIRPα on proximal tubule. (**A**) Fatty acid synthesis genes in normal chow (NC) or high fat diet (HFD) kidney were determined by real-time RT-PCR (n=8). (**B**) The expression of fatty acid synthesis genes was checked in 24 h-recombinant SIRPα (rSIRPα, 500 ng/mL) treated BUMPT cells (n=4). (**C**) Fatty acid uptake was determined in BUMPT cells after rSIRPα (500 ng/mL) treatment. (n=2 for negative control; n=8 for treatment). Values are expressed as a mean ± SEM. Statistical significance was performed using two-tailed unpaired t-test. *P < 0.05, **P < 0.01 NC vs HFD.

Supplementary Table 1. Chronic kidney disease patients' characteristics.

	Non-DM	DM	P Value
**Gender**	24M/38	7M/11	
**Age**	61±2.38	68±2.84	0.1562253
**Serum creatinine (mg/dL)**	8.4±0.66	5.97±0.54	0.064727
**Diabetes**	0/38	11/11	
**Hypertension**	26/38	11/11	

Non-Diabetic CKD Patients

Age	Gender	Race	s-creatinine	Diabetes	hypertension
52	M	Caucasian	8.3	no	yes
45	M	Caucasian	11	no	yes
64	F	Caucasian	8.6	no	no
39	M	Caucasian	14.8	no	no
58	M	Caucasian	10.9	no	yes
48	M	Caucasian	10.1	no	yes
48	M	Caucasian	21.8	no	no
46	F	Caucasian	9	no	yes
46	M	Caucasian	12.8	no	no
56	M	Caucasian	15.3	no	no
53	M	Caucasian	9.1	no	yes
52	F	Caucasian	7.1	no	no
56	F	Caucasian	11.6	no	yes
53	F	Caucasian	9.2	no	yes
64	M	Caucasian	13	no	yes
47	M	Caucasian	11.6	no	yes
60	F	Caucasian	7.1	no	yes
49	F	Caucasian	10.5	no	yes
30	F	African	13.1	no	no
40	M	Caucasian	12.2	no	yes
70	M	Caucasian	4.4	no	yes
60	F	Caucasian	4.4	no	yes
65	F	Caucasian	5.4	no	yes
71	M	Caucasian	5.9	no	yes
83	M	Caucasian	7.2	no	yes

64	M	Caucasian	5.1	no	no
73	F	Caucasian	5.8	no	yes
47	M	Caucasian	6.6	no	yes
76	M	Caucasian	4.5	no	yes
90	F	Caucasian	3.5	no	yes
67	M	Caucasian	4.6	no	no
89	F	Caucasian	4.4	no	no
77	M	Caucasian	6.2	no	yes
69	F	Caucasian	3.1	no	no
71	M	Caucasian	4.5	no	yes
81	M	Caucasian	5.2	no	no
79	M	Caucasian	5.5	no	yes
78	M	Caucasian	4.6	no	yes

Diabetic CKD Patients

Age	Gender	Race	s-creatinine	Diabetes	hypertension
65	F	Caucasian	8.3	yes	yes
79	M	Caucasian	7.1	yes	yes
75	M	Caucasian	5.8	yes	yes
70	M	Caucasian	9.7	yes	yes
71	M	Caucasian	5.1	yes	yes
53	F	Caucasian	5.5	yes	yes
68	F	Caucasian	3.9	yes	yes
68	M	Caucasian	5.3	yes	yes
74	M	Caucasian	3.8	yes	yes
48	F	Caucasian	6.1	yes	yes
74	M	Caucasian	5.1	yes	yes
