# Supplementary figures and images for: Myokine SIRPα exacerbates kidney disease in diabetes
Source: JCI Insight. 2026 Feb 9;11(3):e183392. doi: 10.1172/jci.insight.183392 (PMC12893106; doi:10.1172/jci.insight.183392)

Fig 1E

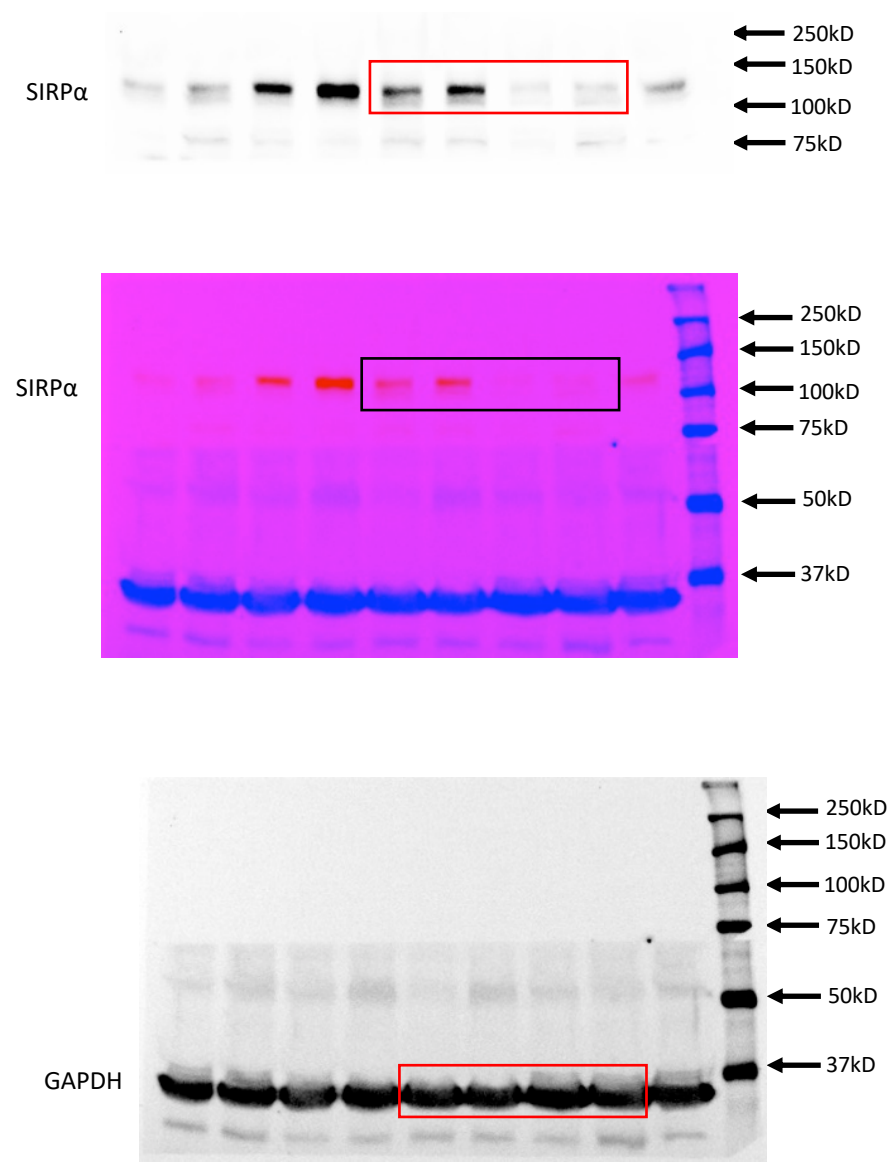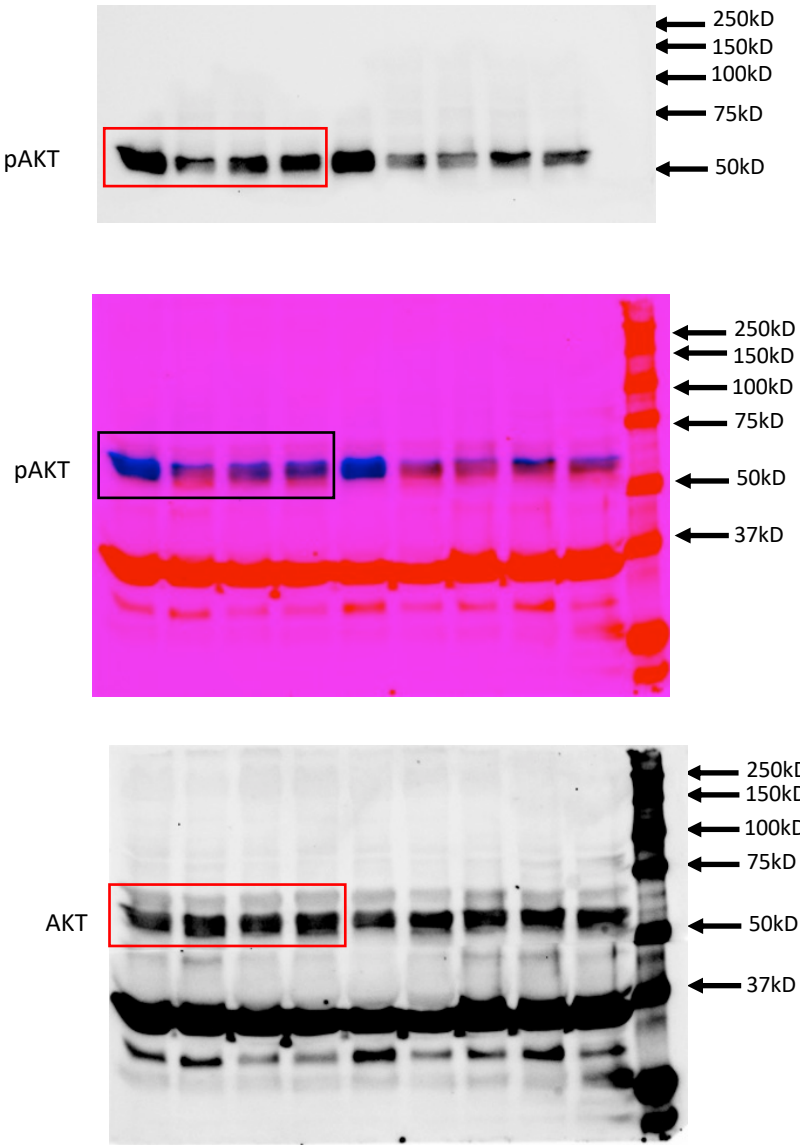

Fig 1F

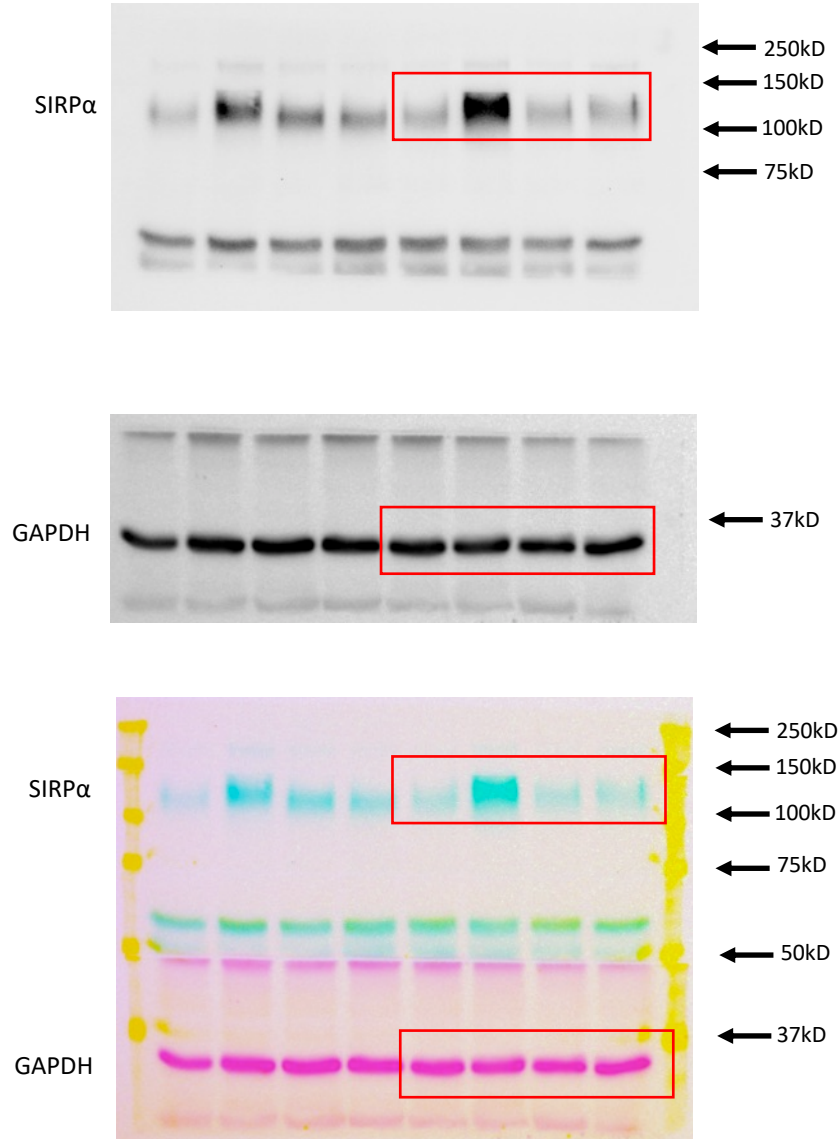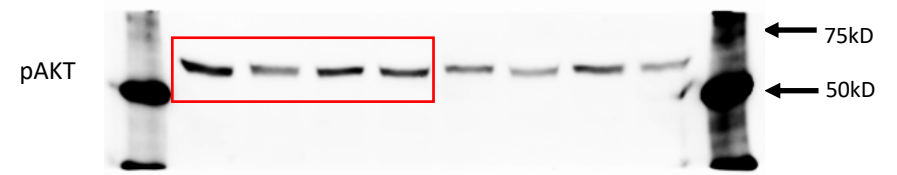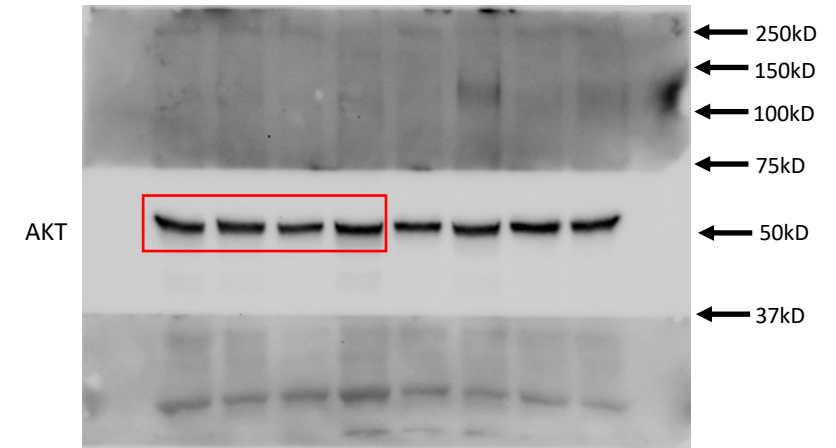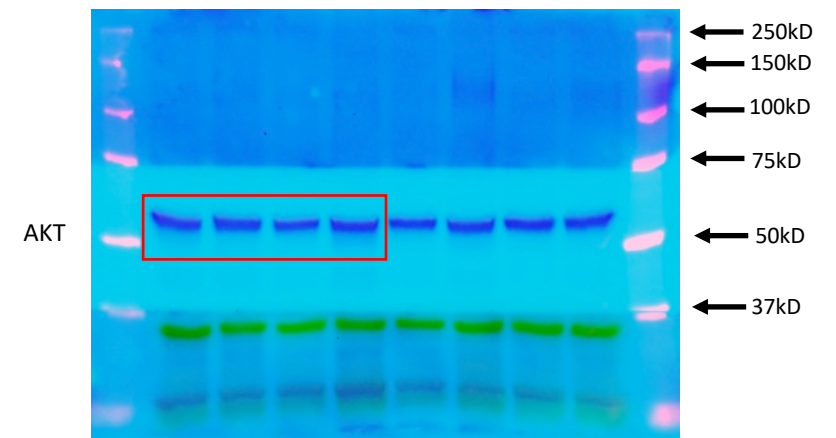

Fig 2B

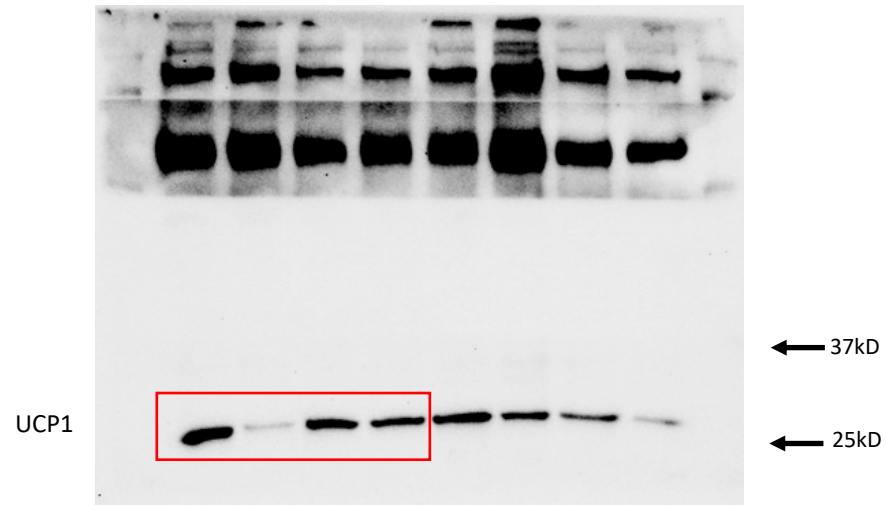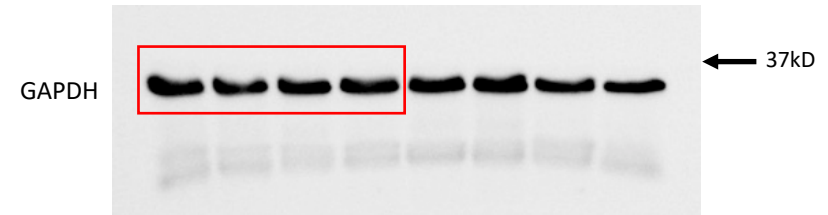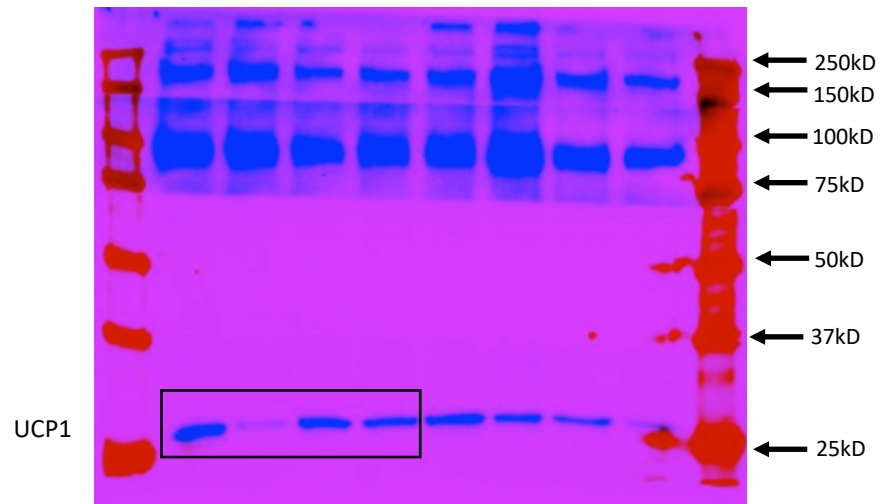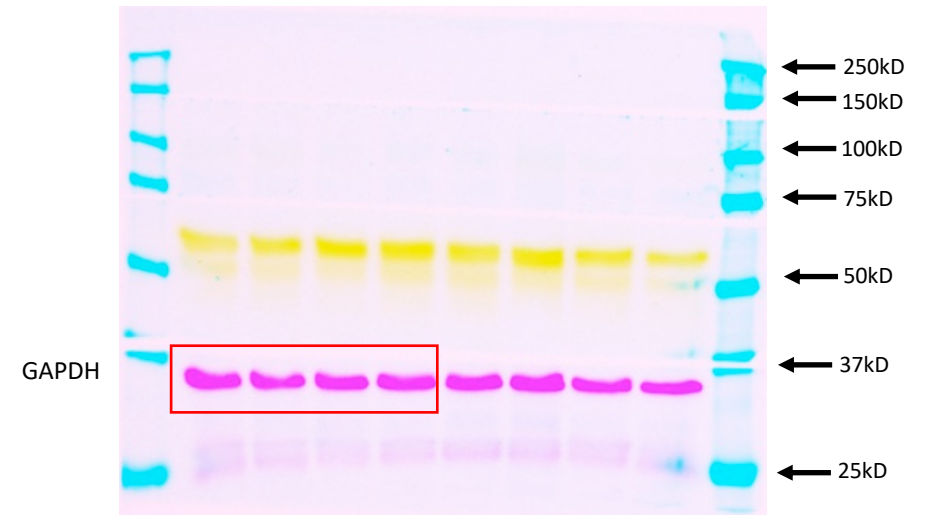

Fig 2E

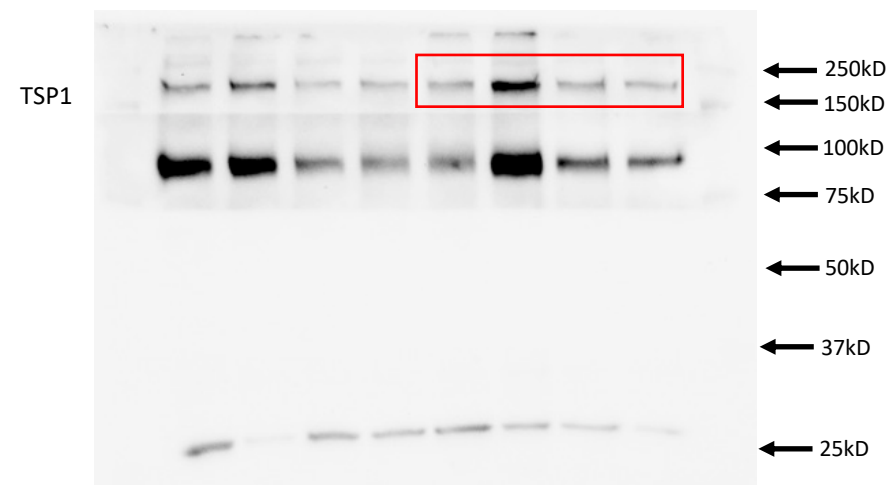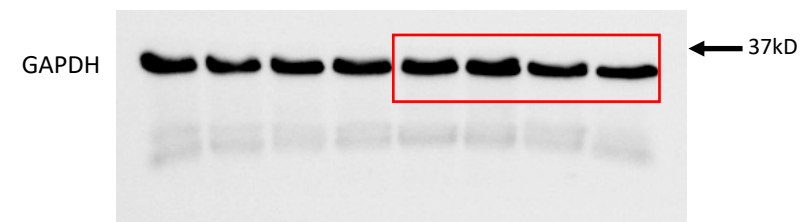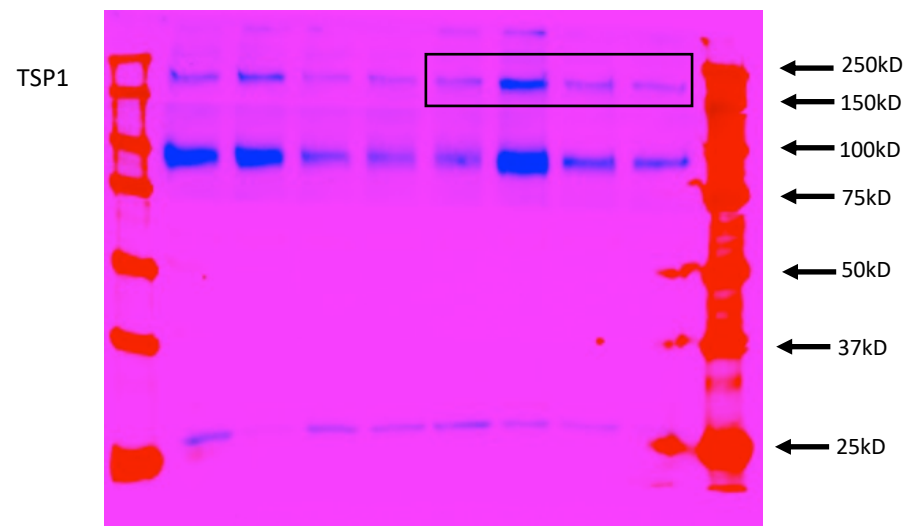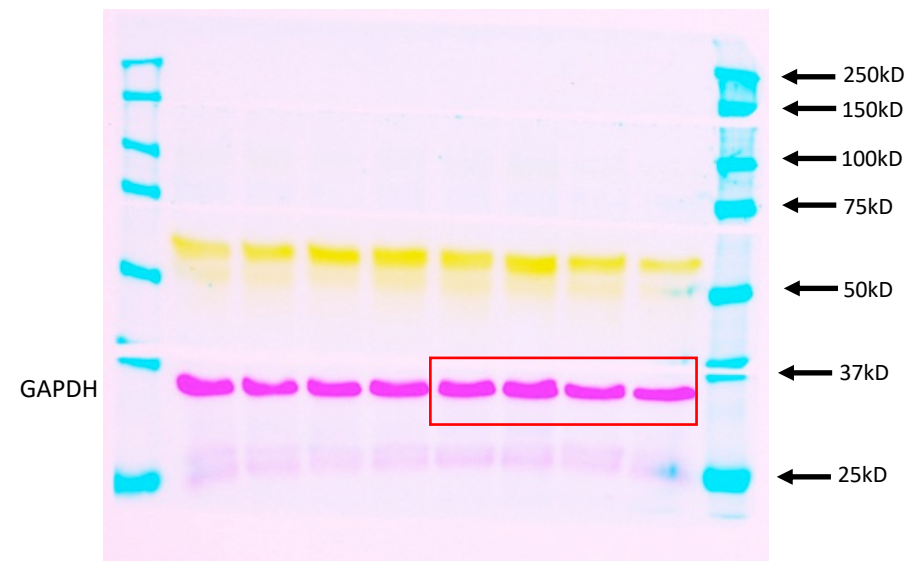

Fig 4H

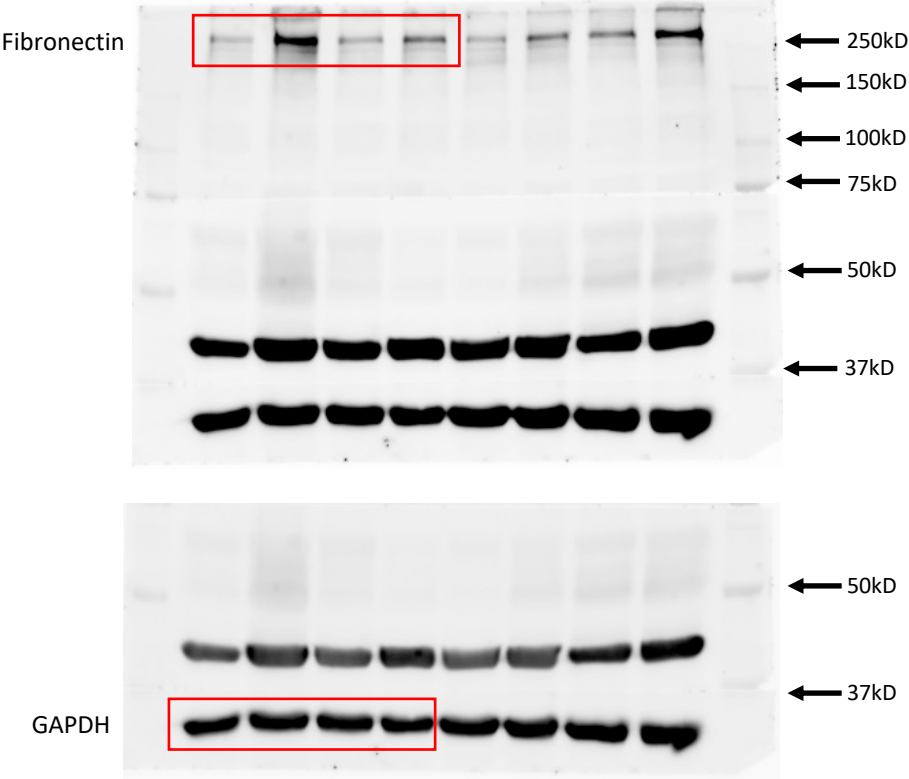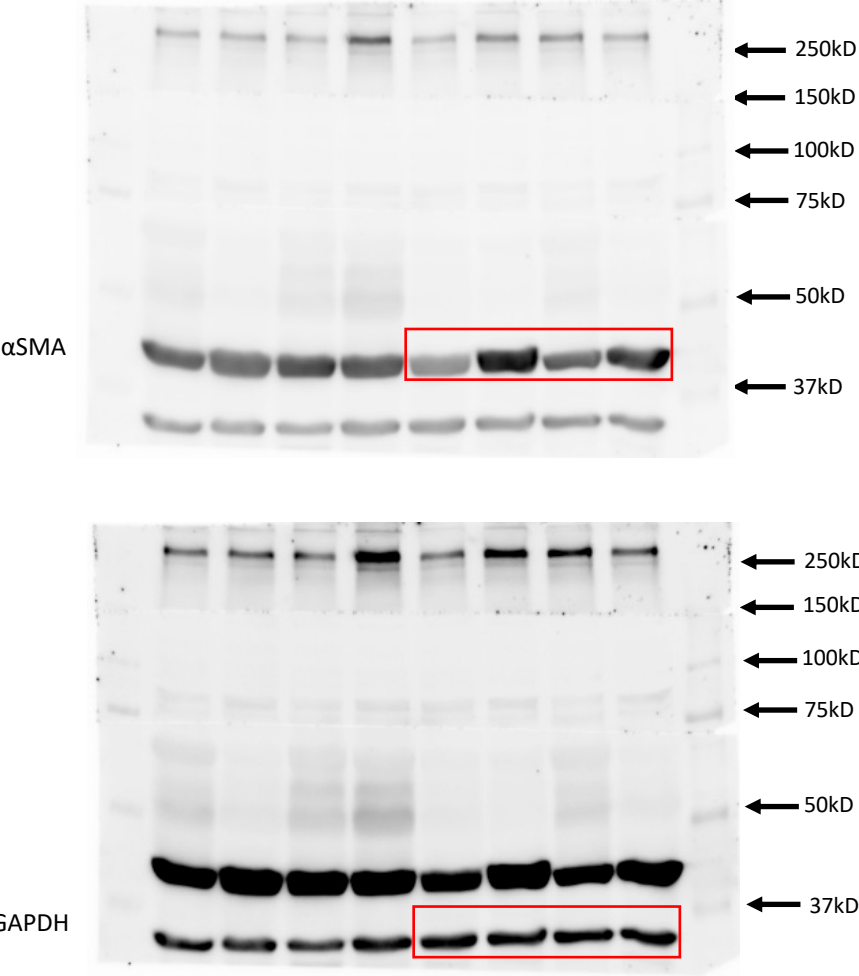

Fig 4K

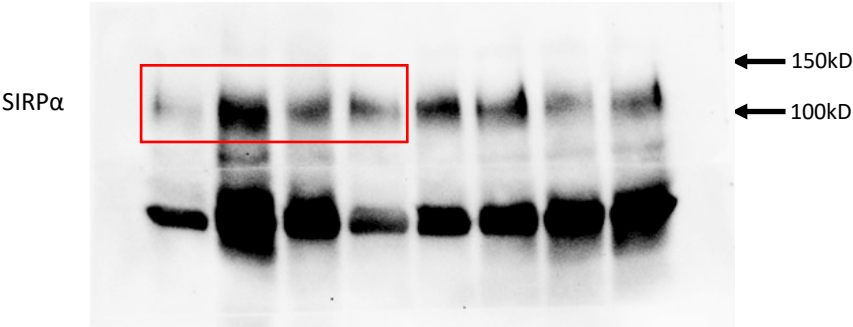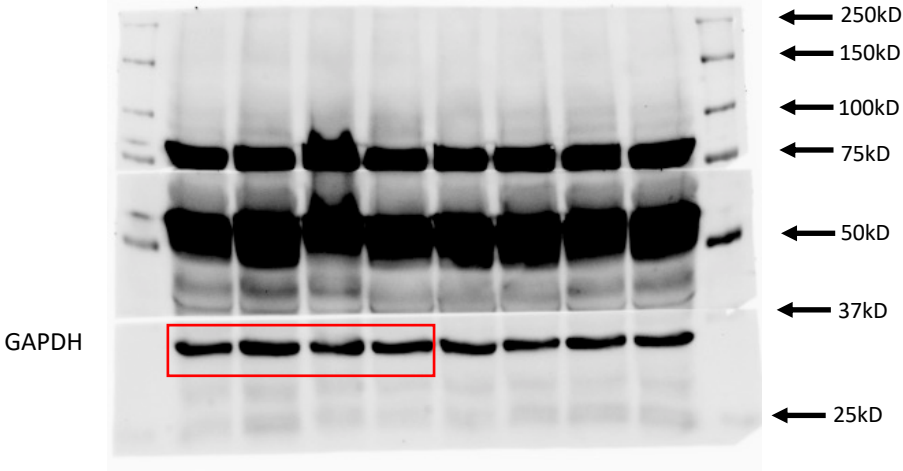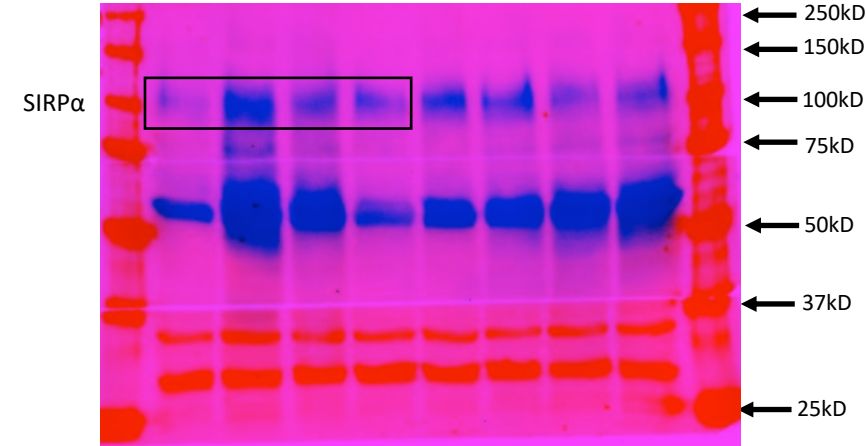

**Fig S1A**

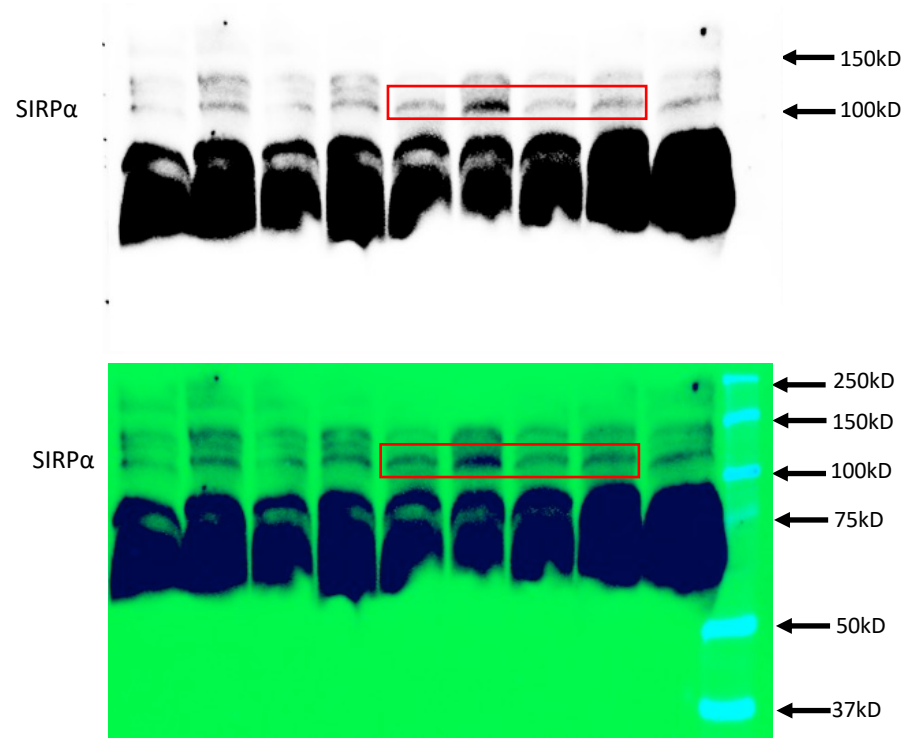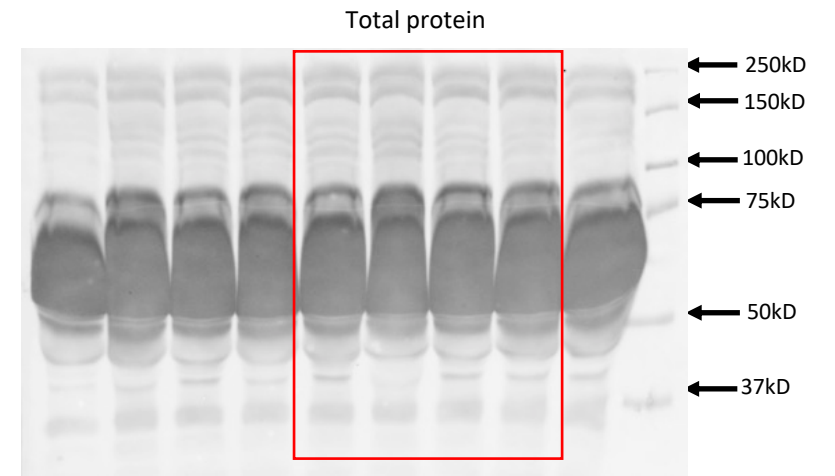

Fig S5D

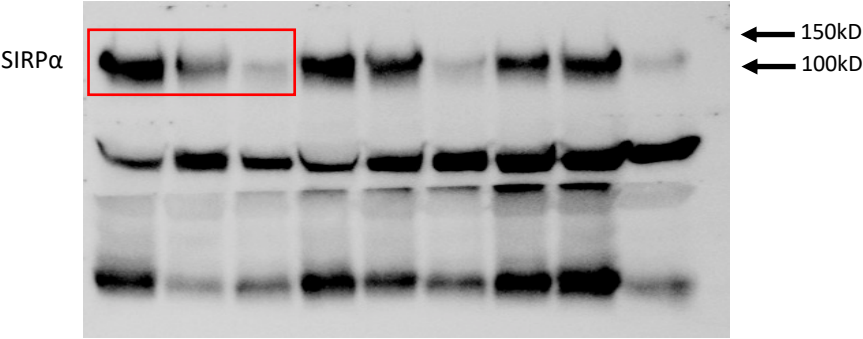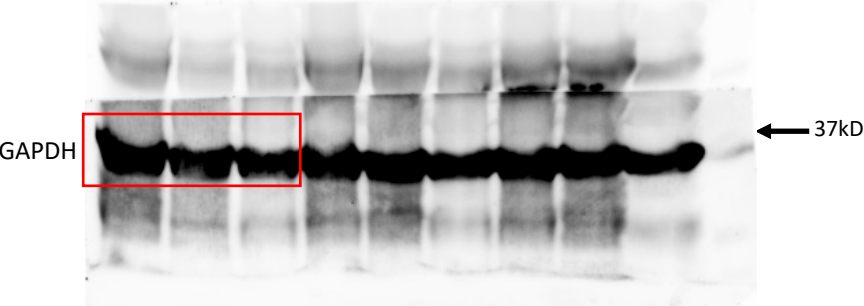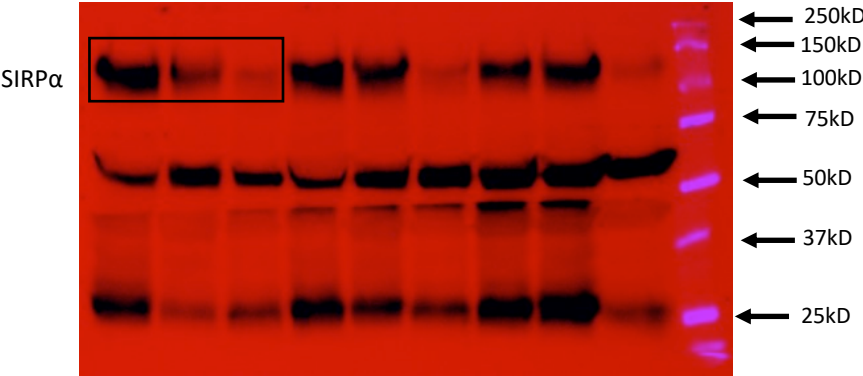

**Fig S5E**

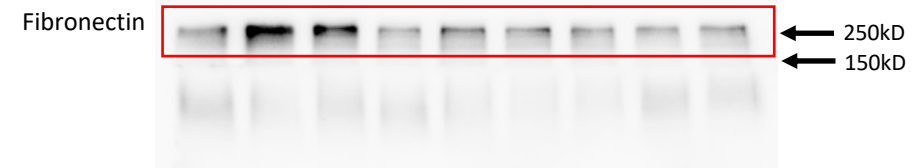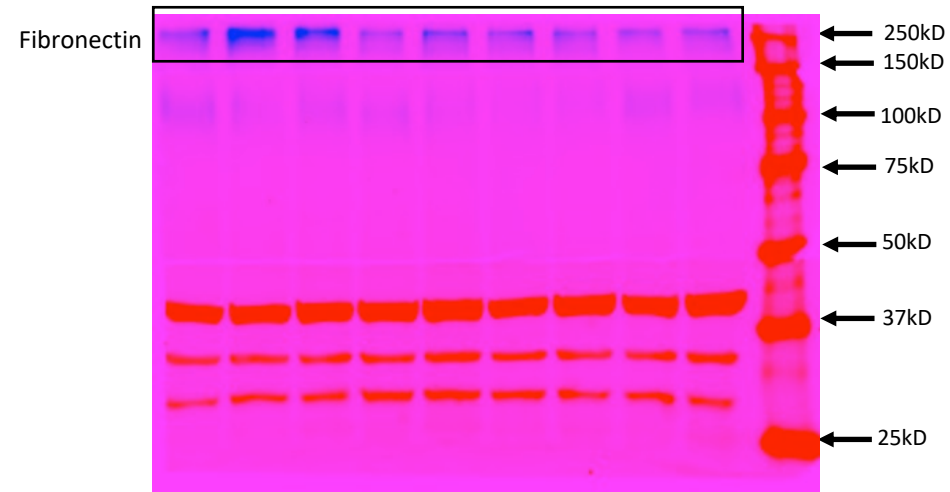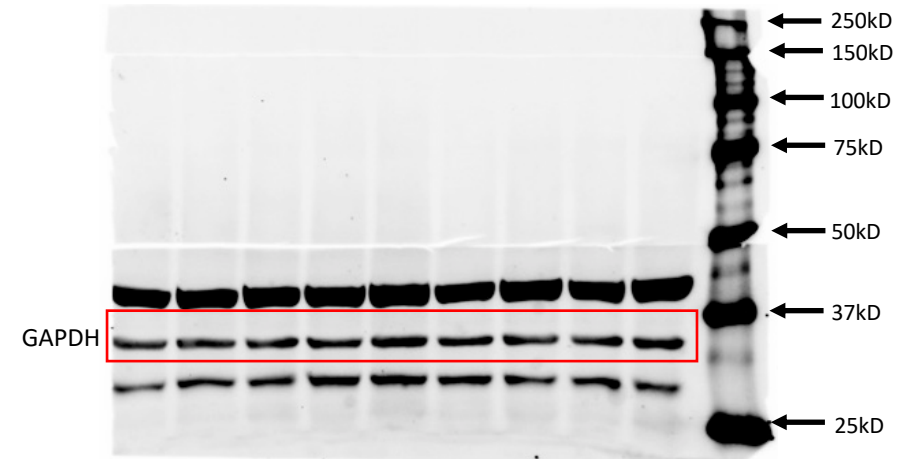

Supplement: Unedited blot and gel images [file jciinsight-11-183392-s291.pdf]
